# Programmed meiotic errors facilitate dichotomous sperm production in the silkworm, *Bombyx mori*

**DOI:** 10.1073/pnas.2520991123

**Published:** 2026-02-25

**Authors:** Leif Benner, Makenzie Richmond, Youbin Xiang, LingSze Lee, Clio B. Hockens, Tianwei Li, Zulin Yu, Dai Tsuchiya, Shengping Huang, Eelco C. Tromer, R. Scott Hawley, Leah F. Rosin

**Affiliations:** 1 Unit on Chromosome Dynamics, Division of Development Biology, *Eunice Kennedy Shriver* National Institute of Child Health and Human Development, National Institutes of Health, Bethesda, Maryland, 20982, USA.; 2 Stowers Institute for Medical Research, Kansas City, Missouri, 64110, USA.; 3 Molecular Genomics Core, *Eunice Kennedy Shriver* National Institute of Child Health and Human Development, National Institutes of Health, Bethesda, Maryland, 20982 USA.; 4 Cell Biochemistry, Groningen Biomolecular Sciences & Biotechnology Institute, Faculty of Science and Engineering, University of Groningen, Linneausborg, Nijenborgh 7, 9747AG Groningen, the Netherlands.

**Keywords:** Spermatogenesis, meiosis, holocentric, silkworm, eupyrene, apyrene, chromosome segregation, RNA-seq, transcription, Major: Biological Sciences, Minor: Genetics Cell Biology

## Abstract

The goal of meiosis is typically to produce haploid gametes (eggs or sperm). Failure to do so is catastrophic for fertility. However, Lepidopteran (moths and butterflies) males produce two sperm morphs: nucleated (eupyrene) sperm and anucleated (apyrene) sperm, both of which are essential for fertilization. The meiotic differences in the two types of spermatogenesis are unclear, and our knowledge of the molecular differences between eupyrene and apyrene spermatogenesis are extremely limited in all systems. The only factor identified as being required for apyrene spermatogenesis is *Sex-lethal* (*Sxl*). Here, we show through cytological analysis of meiotic events that there are several key differences in the genesis of apyrene and eupyrene sperm. Specifically, during meiosis I, apyrene spermatocytes fail to decondense and pair their chromosomes during meiotic prophase I. Telomeres fail to localize to the nuclear periphery, and full-length synaptonemal complex doesn’t form. We also find evidence of an abnormal second cell division during apyrene meiosis. RNA sequencing of both eupyrene- and apyrene-producing testes reveals distinct changes in transcriptional programs, including down-regulation of a myriad of cell division genes during apyrene meiosis. By comparing wildtype and *Sxl*-knockout apyrene testes, we found that *Sxl* isn’t required for regulating the expression of the cell division genes but instead may play a role in blocking hormone signaling from altering testis cell identity. Together, our findings reveal significant insights into two converging molecular pathways that promote the formation of dimorphic sperm in Lepidoptera.

## Introduction

Dichotomous spermatogenesis, or the production of two distinct yet required spermatozoa, is a phenomenon that occurs in many invertebrate species. This was first described in 1902 through the study of spermatogenesis in both a species of snails and moths (1). More recent work has shown that dichotomous spermatogenesis is a conserved spermatogenic program throughout the order Lepidoptera (moths and butterflies). In the context of Lepidoptera, dichotomous spermatogenesis refers to the production of nucleated sperm (eupyrene) and anucleated sperm (apyrene), with both being required for male fertility (2). The production of these two sperm morphs occurs during distinct timepoints in development. Although slight differences exist in the developmental production of these sperm morphs between species, generally, eupyrene sperm are made first during larval development and the switch to apyrene sperm production occurs during the pupal stages (reviewed in (3)).

How eupyrene and apyrene meioses produce normal versus anucleate sperm remains unclear and is largely inferred from cytological studies. Eupyrene spermatocytes follow a conserved, canonical meiotic program (3, 4), whereas apyrene spermatocytes are believed to lack key meiotic features, including homolog pairing, stable synaptonemal complex formation, bivalent assembly, and telomere–nuclear envelope attachment (reviewed in (3)). These defects are thought to disrupt chromosome orientation and lead to non-homologous alignment at metaphase I, unresolved chromosome bridges, chromosome fractionation, and the formation of mini-chromosomes (reviewed in (3)). As a result, daughter cells inherit unequal chromosome content that is further exacerbated in meiosis II, culminating in nuclear loss during sperm maturation (reviewed in (3)). This process, termed nuclear elimination, ejection, or degradation, likely occurs via peristaltic squeezing and produces sperm largely devoid of DNA (5-10). Together, these observations support the hypothesis that the eupyrene to apyrene switch is driven by dysregulation of cell division genes during the larval-to-pupal transition (reviewed in (3)).

While the mechanism regulating the eupyrene to apyrene switch is unclear, two distinct factors have been implicated in driving apyrene spermatogenesis. One proposed regulator is hormone signaling, which is known to drive metamorphosis in insects. Evidence for this is that in many Lepidoptera, transplanting larval testes into pupal tissues, regardless of sex, is sufficient to induce the switch from eupyrene to apyrene spermatogenic programs (11). Also, experimentally inducing precocious pupation is sufficient to induce apyrene spermatogenesis earlier than age-matched controls (12). Therefore, there is likely a systemic hormone signal during development promoting the induction of apyrene spermatogenesis. The precise signal is unknown, however, but is commonly referred to as the apyrene spermatogenesis inducing factor (ASIF).

The second proposed regulator of apyrene sperm production is the RNA-binding protein Sex-lethal (Sxl). There is a vast amount of literature on the function of Sxl in *D. melanogaster*, where it has been well documented to be the primary regulator of somatic sex-determination, dosage compensation, and female germ cell development (13). In *B. mori, S*xl has been shown to be required for apyrene sperm production. Sxl knock-out (KO; *Sxl*^*−*^) testes fail to make functional apyrene sperm, and pupal sperm bundles exist in a described ‘intermediate state’, with a failure to eject nuclei that ultimately results in male-specific sterility (10, 14). Despite these findings, the mechanism through which Sxl regulates the apyrene transition, and if there is a relationship with hormone signaling, have not been described.

In our work on eupyrene and apyrene spermatogenesis in the silkworm *B. mori*, we use updated technologies such as Oligopaint DNA FISH and newly generated antibodies to *B. mori* meiotic proteins to confirm that apyrene spermatocytes fail to execute several conserved meiotic events. Apyrene chromosomes fail to localize telomeres to the nuclear edge, fail to pair homologs, and fail to form proper synaptonemal complex (SC). To investigate the molecular regulation of apyrene spermatogenesis, we perform RNA-sequencing (RNA-seq) and Assay for Transposase-Accessible Chromatin using sequencing (ATAC-seq) on eupyrene and apyrene spermatocytes and show that chromatin accessibility is globally changed and the expression of fundamental cell division genes is broadly perturbed during apyrene spermatogenesis, consistent with our cytological data. Finally, gene expression profiling of *Sxl*^*−*^ testes also provides evidence for a loss of tissue identity in the absence of Sxl, and we find that Sxl is unlikely directly involved in regulating the changes seen in meiotic gene expression between the two sperm morphs. Altogether, our results uncover new cell biology regulating a unique meiotic pathway in moths and provides a testable roadmap for future interrogation of the mechanistic changes that occur between eupyrene and apyrene spermatogenesis.

## Results

### Chromosomes in apyrene spermatocytes fail to decondense, pair, or form a proper synaptonemal complex during prophase I

**Figure 1.**
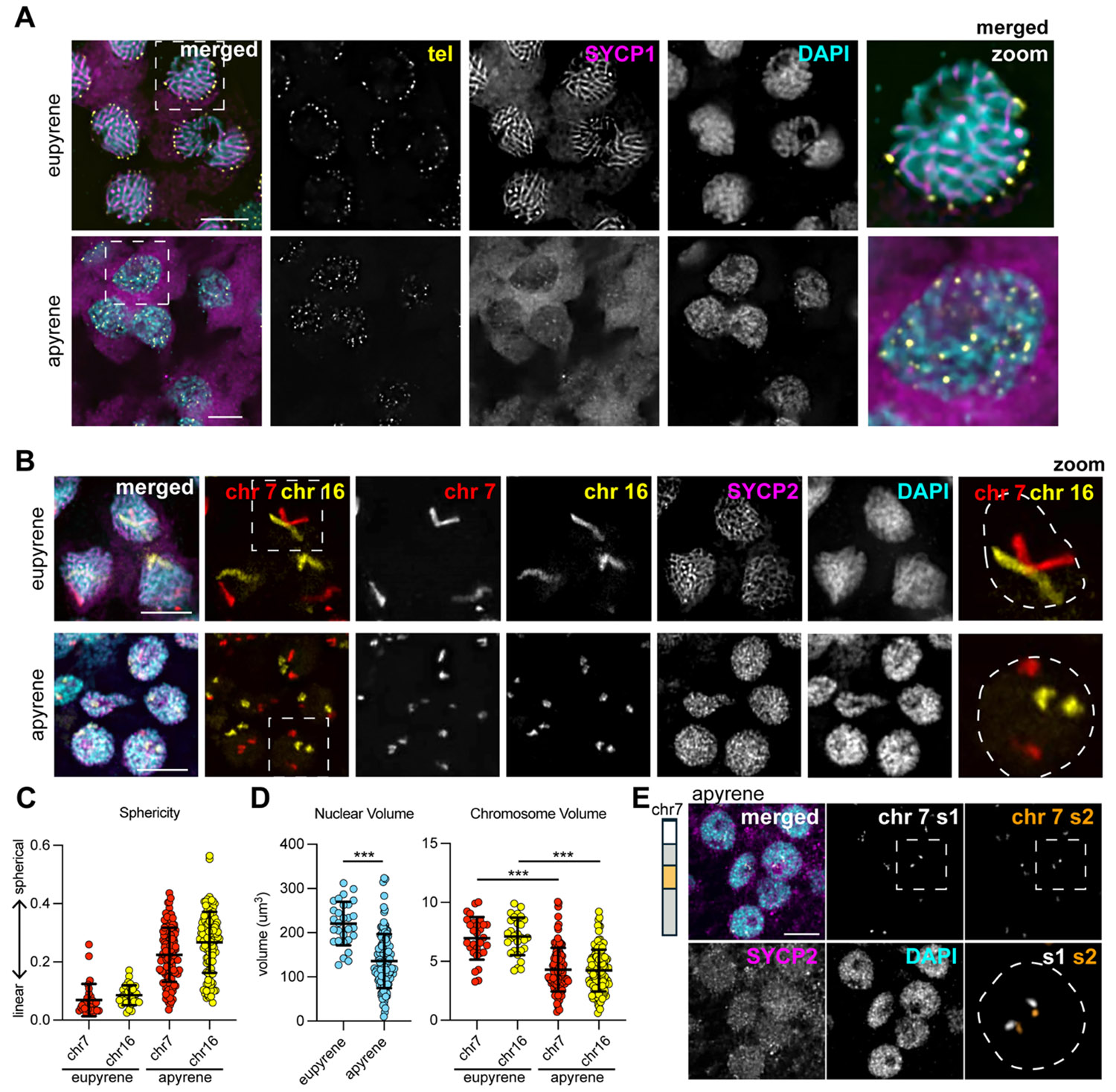
Robust prophase I errors are observed in apyrene spermatocytes. A. Eupyrene (top) and apyrene (bottom) spermatocytes from cryosectioned 5^th^ instar and day 1 pupal testes, respectively. Shown: DAPI (cyan), SYCP1 (magenta), FISH to telomeric repeats (tel; yellow). Scale bars is 5 μm. B. Representative i mages from cryosections labeled with Oligopaint FISH for chr 7 (red) and 16 (yellow) on prophase I eupyrene (5^th^ instar larvae, top) and apyrene (prepupae, bottom) spermatocytes. Unlike eupyrene spermatocytes that form SC and pair homologous chromosomes, apyrene spermatocytes don’t form proper SC threads and don’t pair, as shown through SYCP2 and the Oligopaints. DAPI is shown in cyan and SYCP2 in magenta . Apyrene spermatocyte chromosomes are more condensed than in eupyrene spermatocytes in prophase I, and don’t display thread-like shapes. Scale bar is 5 μm. C. Dot column plot showing the sphericity of chromosomes measured by TANGO (see methods). Sphericity is the ratio between the volume and surface area of an object, where 1 is a perfect sphere. ***p<0.0001; Welch’s T-test. Each dot represents one chromosome from a single replicate. The experiment was repeated in triplicate. D. Left: Dot column plot showing nuclear volume measured by TANGO (see methods). Right: Dot column plot showing volume of Oligopaint FISH as a proxy for chromosome volume. ***p<0.0001; Welch’s T-test comparing eupyrene to apyrene. Each dot represents one nucleus from a single replicate. The experiment was repeated in triplicate. E. Oligopaint stripes chr 7 s1 (white) and chr 7 s2 (orange) on prophase I cryosectioned apyrene testes (day 1 pupa) demonstrate that each homolog remains intact and doesn’t fragment as paints remain together on each homolog. DAPI is shown in cyan and SYCP2 in magenta . Image shows apyrene prophase I spermatocytes from day 1 pupa. Scale bar is 5 μm.

To investigate telomere dynamics, homolog pairing, and SC formation during eupyrene and apyrene meiosis, we first examined prophase I in larval (eupyrene) and pupal (apyrene) testes using a combination of immunofluorescence and Oligopaint DNA FISH (Fig S1A-B). As expected for a canonical meiotic prophase I, imaging experiments using a FISH probe labeling the insect telomeric repeat and IF labeling SC proteins revealed that in eupyrene pachytene, telomeres cluster around the nuclear edge (as determined by the edge of DAPI staining) and the SC forms thread-like fibers between homologs throughout the nucleus (Fig 1A top, S1C-D) (4, 15-17). Conversely, in apyrene prophase I, telomeres never cluster and fail to localize to the nuclear edge (Fig 1A bottom). Additionally, consistent with prior reports, we see that a complete SC fails to form during apyrene meiosis (3), with SYCP1 (transverse filament protein) being essentially absent from nuclei, and lateral element components like SYCP2, SYCP3, and HOP1 being nuclear but not forming classical SC fibers at pachytene (Fig 1A bottom, S2A-B).

Consistent with incomplete SC formation, telomeres fail to pair during apyrene spermatogenesis. *B. mori* has 28 chromosome pairs, predicting ~56 telomere foci when pairing is intact. H owever, apyrene prophase I nuclei consistently contain >80 telomere foci, compared with ~50 in eupyrene prophase I (Fig S2C), indicating widespread telomere pairing defects. Chromosome painting of chromosomes 7 and 16 further revealed major defects in homolog pairing during apyrene prophase I, with two distinct FISH signals per nucleus rather than the single, linear signal seen in eupyrene cells (Fig 1B, S2D). Apyrene chromosomes also appear spherical rather than linear as in eupyrene meiosis, suggesting a failure of the chromosome decompaction normally associated with meiotic entry (4, 18-20; Fig 1C), and consistent with significantly smaller nuclear and chromosome volumes in apyrene compared with eupyrene SYCP2-positive cells (Fig 1D, S2E). Despite these organizational defects, chromosomes are intact and not fragmented, as shown by sub-chromosomal Oligopaint FISH (Fig 1E, S2F). Together, these data demonstrate severe errors in homolog pairing at both telomeres and along the chromosome length during apyrene meiosis I.

### Apyrene metaphase I nuclei harbor unaligned, unlinked univalents

**Figure 2.**
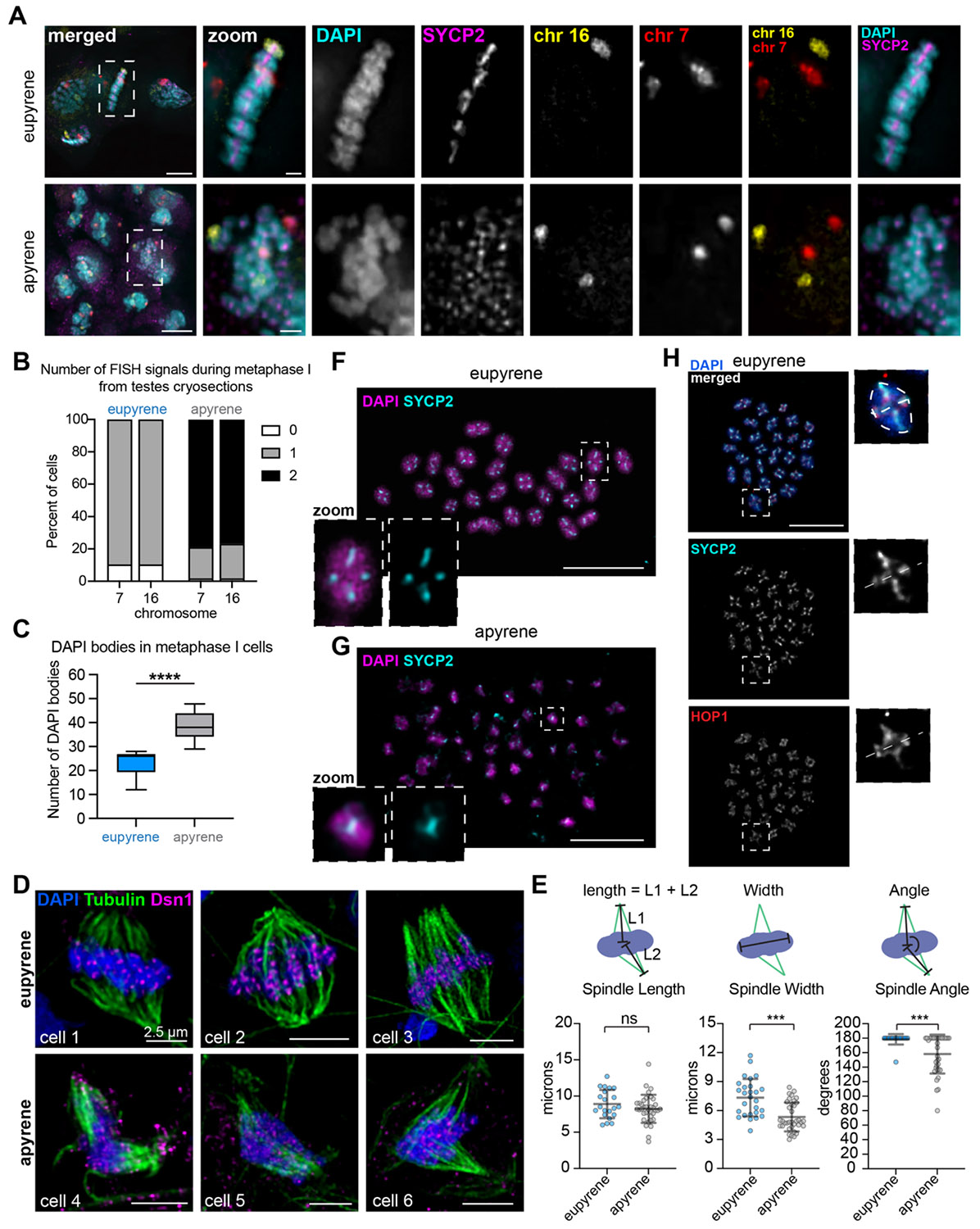
Homologs fail to align and pair at metaphase I. A. Eupyrene (top) and apyrene (bottom) spermatocytes from cryosectioned testes labeled with DAPI (cyan), Oligopaints for chr 7 (red) and chr 16 (yellow), and SYCP2 (magenta). Scale bar is 5 μm in merged (left) and 1 μm for zoomed panels (right). Images are maximum-intensity projections of a deconvolved *z*-series through the selected cell. B. Quantification of the number of Oligopaint FISH signals in metaphase I cells, as shown in A. 90% of metaphase I eupyrene spermatocytes (5^th^ and mature instars combined) have 1 signal at metaphase I (n = 77). Conversely, 76% & 79% (chr 16 and chr 7) of metaphase I apyrene spermatocytes (day 3 pupa) have 2 signals at metaphase I (n = 103). C. Quantification of the number of chromosome masses in eupyrene and apyrene metaphase I spreads. p < 0.0001; unpaired t-test (Kolmogorov-Smirnov test). D. Representative images of IF on metaphase cells from eupyrene (top) and apyrene (bottom) testes chromosomes spreads. DAPI is shown in blue, tubulin in green, and Dsn1 in magenta. Scale bar is 2.5 μm. Spindles may appear slightly open due to loss of aster microtubules and centrosomes during the spreading process. E. Top: Schematic of measurements taken for bottom graphs. Bottom: Dot plot showing the spindle length (left), spindle width (middle), and spindle angle (right) of eupyrene (blue) and apyrene (gray) metaphase I cells. ***p ≤ 0.0001. Unpaired t-test with Welch’s correction. F. Metaphase I spread from 5^th^ instar larval testes showing that eupyrene spermatocytes have approximately 28 bivalents, and SYCP2 (cyan) remains between homologs and between sister chromatids. Bottom: Zoom of indicated chromosome. DAPI is shown in magenta. Scale bar is 10 μm. G. Metaphase I spread from day 7 pupal testes showing that apyrene spermatocytes have more than 28 DAPI bodies, and SYCP2 (cyan) remains between sister chromatids at metaphase I. Bottom: Zoom of indicated chromosome. DAPI is shown in magenta. Scale bar is 10 μm. H. Metaphase I spread from 5^th^ instar larval testes showing that eupyrene spermatocytes have approximately 28 bivalents, and SYCP2 (cyan) and HOP1 (red) remain between homologs and between sister chromatids. Right: Zoom of indicated chromosome. DAPI is shown in blue. Scale bar is 10 μm.

Previous cytological studies on apyrene meiosis in *B.mori* and other Lepidoptera have indicated errors with metaphase chromosome alignment and chromosome segregation during anaphase of meiosis I (3, 21-23). To verify these findings, we visualized chromosomes in spermatocytes at metaphase I in both eupyrene and apyrene meiosis. In eupyrene metaphase I, we observed that chromosomes are tightly aligned at the metaphase plate (Fig 2A). Additionally, FISH with chromosomes 7 and 16 Oligopaints in eupyrene metaphase I cells show homologs in bivalent structures as indicated by a single FISH signal per chromosome (Fig 2A). This agrees with previous reports in *B. mori* (4) and is consistent with a canonical meiotic program. Conversely, in apyrene metaphase I nuclei, we found severe chromosome alignment defects without a clear “metaphase plate” (Fig 2A), similar to what was observed in prior studies. In line with our analyses of prophase I pairing, FISH with chromosomes 7 and 16 Oligopaints in apyrene metaphase I nuclei yields two distinct FISH signals for each probe, demonstrating that chromosomes form univalent structures at apyrene metaphase I instead of being linked as bivalents (Fig 2A-B, S2G). Additional evidence for unlinked homologs at metaphase I comes from chromosome spreads, which show a median of 26 distinct DAPI bodies at eupyrene metaphase I, but 38 distinct DAPI bodies in apyrene metaphase I (Fig 2C).

We also observed differences in spindle morphology at metaphase I in apyrene meiosis compared to eupyrene meiosis. Apyrene metaphase I is characterized by more bent and narrow spindle , compared to the linear, broad spindles at eupyrene metaphase I (Fig 2D-E). Additionally, the kinetochore protein Dsn1 (part of the Mis12 complex) is broadly mislocalized based on immunofluorescence staining during apyrene meiosis compared to the distinct kinetochore foci observed on the aligned bivalents in eupyrene meiosis (Fig 2D). All of these results are consistent with previous reports of chromosome segregation errors in apyrene meiosis (3, 24), and suggest a plethora of errors in early meiotic processes.

### Lateral elements of the SC persist through metaphase I in eupyrene and apyrene spermatocytes

Our imaging of eupyrene and apyrene metaphase I spermatocytes not only establish that homologs are unlinked at metaphase I in apyrene meiosis, but the images also reveal a curious localization pattern of SC proteins. For example, in our cryosectioned testes, SYCP2 persists on chromosomes at metaphase I in both eupyrene and apyrene meiosis (Fig 2A). To further investigate SC persistence at metaphase, we performed testes spreads followed by immunofluorescence of SYCP1, SYCP2, HOP1, and REC8 (Fig 2F-H, S3, S4). While it is expected that REC8 remains associated with chromosomes at metaphase I (25, 26), we unexpectedly found that other lateral components of the SC (HOP1 and SYCP2) also remain associated with chromosomes through metaphase I in both eupyrene and apyrene meiosis (Fig 2F-H, S3, S4). In eupyrene meiosis, where homologs form bivalents, these factors not only localize between homologs at metaphase I, but also between sister chromatids (Fig 2F, 2H, S3, S4), explaining their persistence on univalents at apyrene metaphase I (Fig S4). Conversely, SYCP1 is gone by metaphase I in eupyrene meiosis (and is entirely absent in apyrene meiosis; Fig S3-S4). The persistence of these proteins on apyrene univalents and their localization between sisters and homologs at eupyrene metaphase I suggests that along with REC8 cohesin, lateral SC elements may be involved in sister chromatid associations during both eupyrene and apyrene meiosis I.

### Apyrene spermatogenesis consists of two abnormal meiotic division

**Figure 3.**
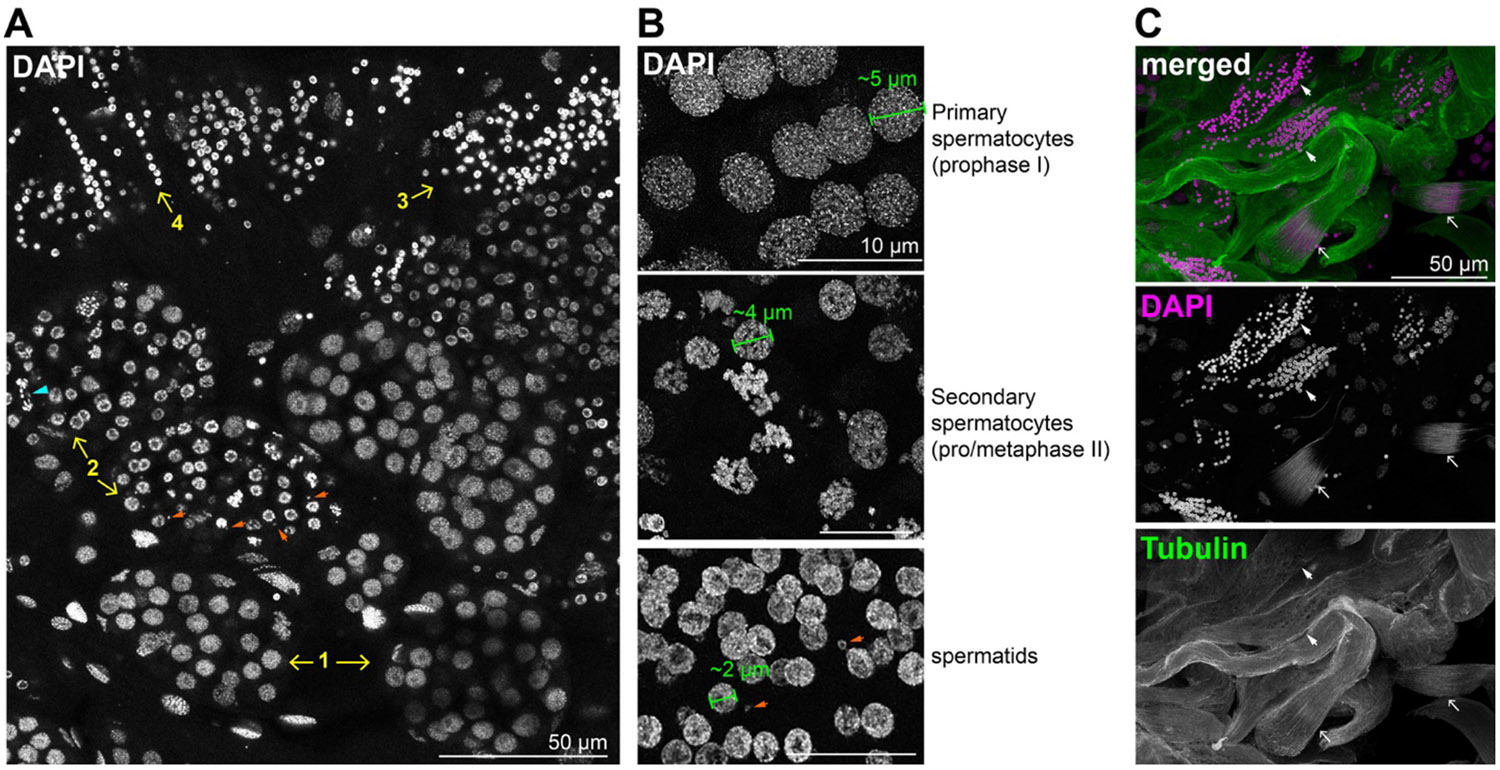
Apyrene meiosis has an abnormal meiosis II A. DAPI staining on a whole-mount WT pupal testis. 1) Cysts of primary spermatocytes in prophase I. 2) Cysts of secondary spermatocytes in prophase/metaphase II. 3) Cyst of round spermatids. 4) Round spermatids aligning as part of spermiogenesis. Orange arrowheads indicate prophase II cells with micronuclei, indicating a prior round of failed chromosome segregation. Cyan arrowhead indicates anaphase II cell with lagging chromosomes. Scale bar is 50 μm. B. Zoomed in views of cells from pupal testes. Top: Primary spermatocyte nuclei. Middle: Secondary spermatocyte nuclei. Bottom: Round spermatids. Green bars indicate nuclear diameter for that specific cell type. Orange arrowheads indicate micronuclei. Scale bars are 10 μm. C. Sperm bundles from WT pupal testes. White arrows indicate normal eupyrene sperm bundles. White arrowheads indicate normal apyrene sperm bundles prior to nuclear loss. DAPI is shown in magenta and tubulin in green. Scale bar is 50 μm.

In apyrene spermatogenesis, not only did we observe an abnormal meiosis I division, but we found evidence of an abnormal meiosis II division before round spermatid nuclei align into apyrene sperm bundles (Fig 3A-B). After apyrene meiosis I, secondary spermatocytes often contain micronuclei, likely due to lagging chromosomes and the inaccurate segregation during meiosis I (Fig 3A). Abnormal chromosome segregation was also observed during anaphase II (Fig 3A), and round spermatids that formed following this division also contain micronuclei (Fig 3A-B). No micronuclei were observed in either secondary spermatocytes or round spermatids in eupyrene meiosis (Fig S5). Additionally, in eupyrene spermatogenesis, round spermatid nuclei eventually undergo a histone-to-protamine transition resulting in needle-shaped nuclei, whereas round spermatids in apyrene meiosis remain large, round, and aligned along microtubule bundles prior to nuclear elimination by peristaltic squeezing, which occurs just before spermatid individualization in the adult gonad (5-10, 27, 28) (Fig 3C and S5).

### Genes required for homolog pairing and synapsis are downregulated during apyrene spermatogenesis

**Figure 4.**
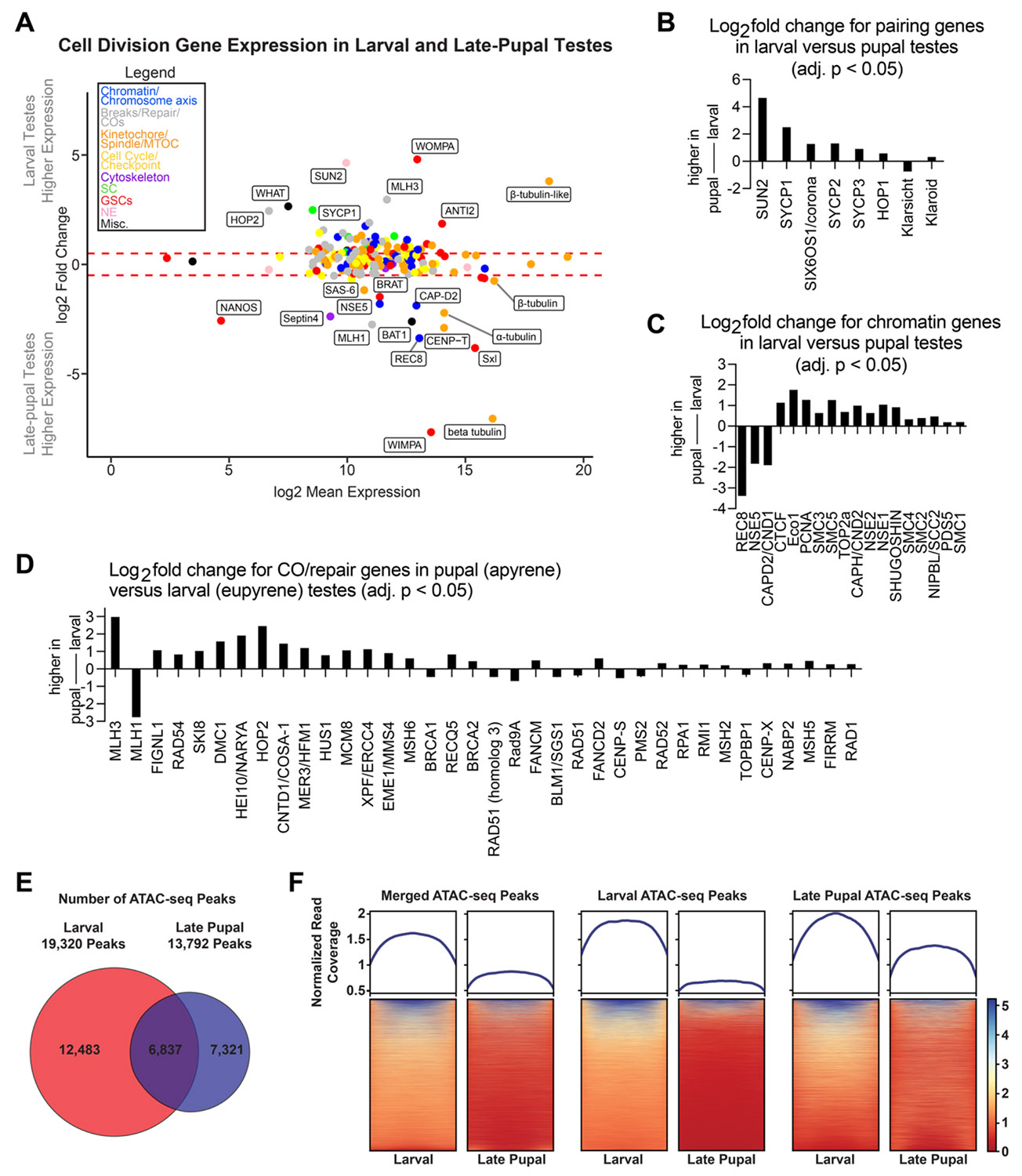
Cell division genes are misregulated during apyrene meiosis A. RNA-seq log2 fold change values of cell division genes between WT larval and late-pupal testes. Select genes and their cell cycle roles [chromatin/chromosome axis (blue), breaks/repair/COs (gray), kinetochore/spindle/MTOC (orange), cell cycle/checkpoint (yellow), cytoskeleton (purple), synaptonemal complex (SC, green), GSCs (germline stem cells, red), nuclear envelope (NE, pink) and miscellaneous (black)] are indicated in the legend. B-D. Bar graphs showing the log2 fold change (Y-axis) for genes (X-axis) that are significantly differentially expressed (adj.p < 0.05) between larval and pupal testes. B = pairing, cytoskeletal and SC genes. C = chromatin genes. D = crossover (CO)/repair genes. E. Number of significant unique and overlapping ATAC-seq peaks in larval and late-pupal testes. F. Normalized read coverage of ATAC-seq peaks in larval and late-pupal testes for merged peaks (intersection of larval and late-pupal ATAC-seq peaks), larval-specific ATAC-seq peaks, and late-pupal-specific ATAC-seq peaks. Heatmap indicates the density of normalized read coverage.

Our cytological analyses confirmed that apyrene spermatocytes fail to properly execute hallmark meiotic events such as telomere clustering, homolog pairing, and metaphase I chromosome alignment. We found support for a disruption of meiotic prophase I pathways in apyrene spermatogenesis: SYCP1 protein is absent from apyrene spermatocyte nuclei, although HOP1, SYCP2, and SYCP3 remain present on sister chromatids. Therefore, we wanted to directly assay meiotic gene expression in eupyrene and apyrene spermatocytes using RNA-sequencing (RNA-seq). To this end, we generated RNA-seq libraries from larval (eupyrene) and late-pupal (apyrene) testes and their somatic tissue counterparts (see supplemental methods; Fig S6). We first checked the expression of genes with previously reported biases in expression: *vasa* (*vlg*, larval testes-enriched), *wompa* (eupyrene-enriched), and *wimpa* (apyrene - enriched) (29, 30). Consistently, we found that *vasa* has significantly higher expression in larval testes compared to somatic tissues, and *wompa* and *wimpa* are specifically enriched in eupyrene (larval) and apyrene (pupal) testes, respectively (Fig 4A, Datasets S1, S2).

Within our RNA-seq, we focused on the expression of previously identified *B. mori* meiotic genes (17) and newly identified *B. mori* orthologs of meiotic, mitotic, and cell cycle genes (referred to collectively as “cell division genes”) (31, 32) (Dataset S2). A total of 214 genes made up our cell division genes list (Dataset S2). This list includes genes for previously unidentified orthologs of critical meiosis proteins, including the central element SC protein SIX6OS1, several different SUN and KASH domain proteins, and the cohesion-related proteins REC8 and Shugoshin (Dataset S2).

We checked if any of these genes were differentially expressed between larval (eupyrene) and late-pupal (apyrene) testes. We found that 32% had higher expression in larval testes, 57% were not differentially expressed, and 11% were higher in late-pupal testes (Fig 4A, Fig S6E; Datasets S1, S2). Among those downregulated in pupal apyrene meiosis were many SC proteins. *Sycp1* showed a five -fold larval enrichment (log2FC = 2.51, padj = 1.14e-30), *sycp2* had greater than two -fold higher larval enrichment (log2FC = 1.28, padj = 1.96e-19), *sycp3* had more than 1.5-fold larval bias (log2FC = 0.90, padj = 2.42e-14), and *hop1* showed a one-fold larval increase (log2FC = 0.47, padj = 1.34e-5) (Fig 4B).

Along with SC proteins being downregulated in late-pupal apyrene testes compared to larval eupyrene testes, we found that the LINC complex *sun2* (33) had a striking 25-fold larval enrichment (log2 fold change = 4.66, padj = 9.07e^−81^) (Fig 4B and Dataset S1). The LINC complex tethers telomeres to the nuclear envelope at meiotic entry and is essential for telomere clustering in many systems (33). A reduction in stable LINC complex during apyrene spermatogenesis is highly consistent with the telomere dispersal we observed cytologically. No other nuclear envelope proteins, such as lamins or nuclear porins, were significantly differentially expressed, suggesting that broad defects in the expression of nuclear envelope genes cannot explain the cytologically observed meiotic phenotypes.

In addition to gene expression changes in testes, we measured cell division gene expression differences between larval testes and larval ovaries (which are achiasmatic (34, 35)). We found that 46% of cell division genes were testes-enriched, 32% were unchanged, and 22% were ovary-enriched (Fig S7A, Dataset S2). Of the 47 DSB/repair/CO genes, 89% were differentially expressed between larval testes and ovaries (Fig S7B), with Spo11, Hei10, and MLH3 being strongly reduced in ovary. 77% of DSB/repair/CO genes were also differentially expressed between larval and pupal testes (Fig S7C), with most being downregulated in pupae.

To test whether these reductions in cell division genes reflected general developmental regulation, we examined somatic tissue as well. Only 7% of cell division genes were downregulated in pupal somatic tissue compared to larval (Fig S6B-D), indicating that the robust decline in cell division gene expression is likely limited to testes.

### Genes involved in chromosome compaction and axis formation are upregulated during apyrene meiosis

To more broadly investigate changes in gene expression in testes during this developmental transition, we conducted gene ontology (GO) term enrichment analysis on the entire set of 3,682 genes that were significantly upregulated in larval eupyrene testes compared to late-pupal apyrene testes (Dataset S1). A single GO term “meiotic nuclear division”, and two cellular component GO terms, “condensed chromosome” and “synapse” (as in nervous system, not chromosome pairing), were considered enriched in larval compared to pupal expression datasets (Dataset S3), supporting a broad reduction of meiotic and cell division gene expression in pupal testes.

Although we found that many meiosis-associated genes decrease in expression in late-pupal testes, this wasn’t the case for all meiosis-associated genes. For example, the meiosis-specific cohesin subunit *rec8* showed greater than ten-fold increased expression in late-pupal apyrene testes compared to larval eupyrene testes (Fig 4A, 4C; Datasets S1 and S2). REC8 cohesin forms the basis of chromatin loops during meiosis and is a major component of the SC lateral elements, facilitating chromosome reorganization at the start of meiosis (36). We also observed increased expression of a condensin I subunit, *capd2*, and the kinetochore protein *cenp-t* (Fig 4A, 4C). Condensin I is required for prophase I cohesin retention in *C. elegans* (37) and CENP-T co-localizes with cohesin along the chromosome axis at pachytene in moths (38). I ncreased expression of these factors during prophase I in apyrene meiosis could lead to smaller chromatin loops and hyper-compacted chromosomes, consistent with our observation that chromosomes fail to properly decondense at meiotic entry in apyrene spermatocytes.

While *rec8* is significantly increased in pupal testes compared to larval testes, SMC1 and SMC3 cohesin subunits were slightly reduced in pupal testes compared to larval testes, as was the cohesin regulator NIPBL (Fig 4C). WAPL wasn’t significantly different between larval and pupal testes (Dataset S1, S2). However, since all of these cohesin factors except REC 8 are not meiosis- specific (and we did not find evidence of a RAD 21L ortholog in *Bombyx*), the lack of coordinated changes in cohesin-related proteins may be due to essential cohesin functions in somatic cells and interphase functions of cohesin (39). Consistent with this idea, RAD 21, the somatic counterpart to REC 8, isn’t significantly differentially expressed between larval and pupal testes (Dataset S1, S2).

We also identified a gene annotated as one of two putative *cenp-e* genes (LOC101738413) that is significantly upregulated during apyrene meiosis (Dataset S1, S2). CENP-E is a kinesin motor protein that stabilizes microtubule-kinetochore attachments and is part of the spindle assemble checkpoint. However, d espite this annotation, the protein shares minimal homology with other CENP-E proteins and lacks both the N-terminal motor and C-terminal microtubule-binding domains (40). psiBLAST and HMMER analyses identified no clear orthologs outside insects (41-43), while HHpred suggests domains associated with flagellar motor function (44, 45; Dataset S4), consistent with a role in sperm motility, the primary function of apyrene sperm (3). This gene is not expressed in larval ovary (Fig S7) and we therefore renamed it *bat1* (*B*ombyx *A*pyrene *T*estes factor 1; Fig 4A). We instead identified LOC101743227 as the most likely true *cenp-e* ortholog.

To investigate whether the increase in REC8 is associated with changes in chromatin organization, such as the increased compaction in apyrene spermatocytes described above, we performed ATAC-seq (Assay for Transposase-Accessible Chromatin using sequencing; (46)) in both whole larval and whole late-pupal testes to look at chromatin accessibility. ATAC-seq analysis indicated that there were 19,320 significantly called peaks in larval testes compared to only 13,792 significantly called peaks in late-pupal testes (Fig 4E-F, S8A), consistent with less compact and more accessible chromatin in eupyrene meiosis. Additionally, the relative accessibility (or peak height) is higher in larval than pupal testes (Fig 4E-F, S8A), further indicating a global increase in accessibility in larval testes. The notable exception to this is the increased accessibility at the promoters of genes that become upregulated in pupal testes/apyrene meiosis, such as *bat1* and the RNA-binding protein *Sex-lethal* (*Sxl*) (Fig S8B-C).

### The misregulation of cell division-associated genes in apyrene spermatocytes is largely independent of *Sex-lethal*

The RNA binding protein *Sex-lethal* (*Sxl*) is required for the eupyrene to apyrene transition in *B. mori* (10, 14). Without functional Sxl protein, eupyrene spermatogenesis proceeds normally, but atypical apyrene sperm are produced (14). However, the role of *Sxl* in the transition from eupyrene to apyrene meiosis is unclear. To dissect the role of Sxl in *B. mori* spermatogenesis, we first investigated *Sxl* expression in wildtype (WT) tissues. For this analysis, we added a mid-pupal timepoint for both testes and soma to better distinguish between early and late developmental roles of Sxl. Our RNA-seq analyses show that *Sxl* expression is low in larval testes and significantly increases in both the mid- (log2 fold change = 3.3, padj = 3.9e^−39^) and late-pupal (log2 fold change = 3.8, padj = 1.6e^−82^) testes stages (Fig 4A, Fig S9). There are two *Sxl* isoforms enriched in the testes throughout development. The larger *Sxl* isoform has similar levels in both larval and pupal testes. In contrast, the smaller *Sxl* protein isoform increases towards the end of larval development and remains high throughout the pupal stages (10, 14).

Previous reports on *B. mori Sxl* suggest that *Sxl* utilizes a single promoter with an alternative splicing decision which results in the large and small protein isoforms (10, 14). However, our RNA-seq data show evidence for an alternative downstream promoter, which was further confirmed with a *de novo* transcript assembly, that splices into exon 3 of *Sxl* thus encoding a downstream start codon which would be consistent with the smaller Sxl protein isoforms (Fig S9A). This promoter is utilized almost exclusively for *Sxl* expression in mid- and late-pupal tissues, consistent with the increase in the smaller Sxl isoform during the pupal stages (10, 14).

**Figure 5.**
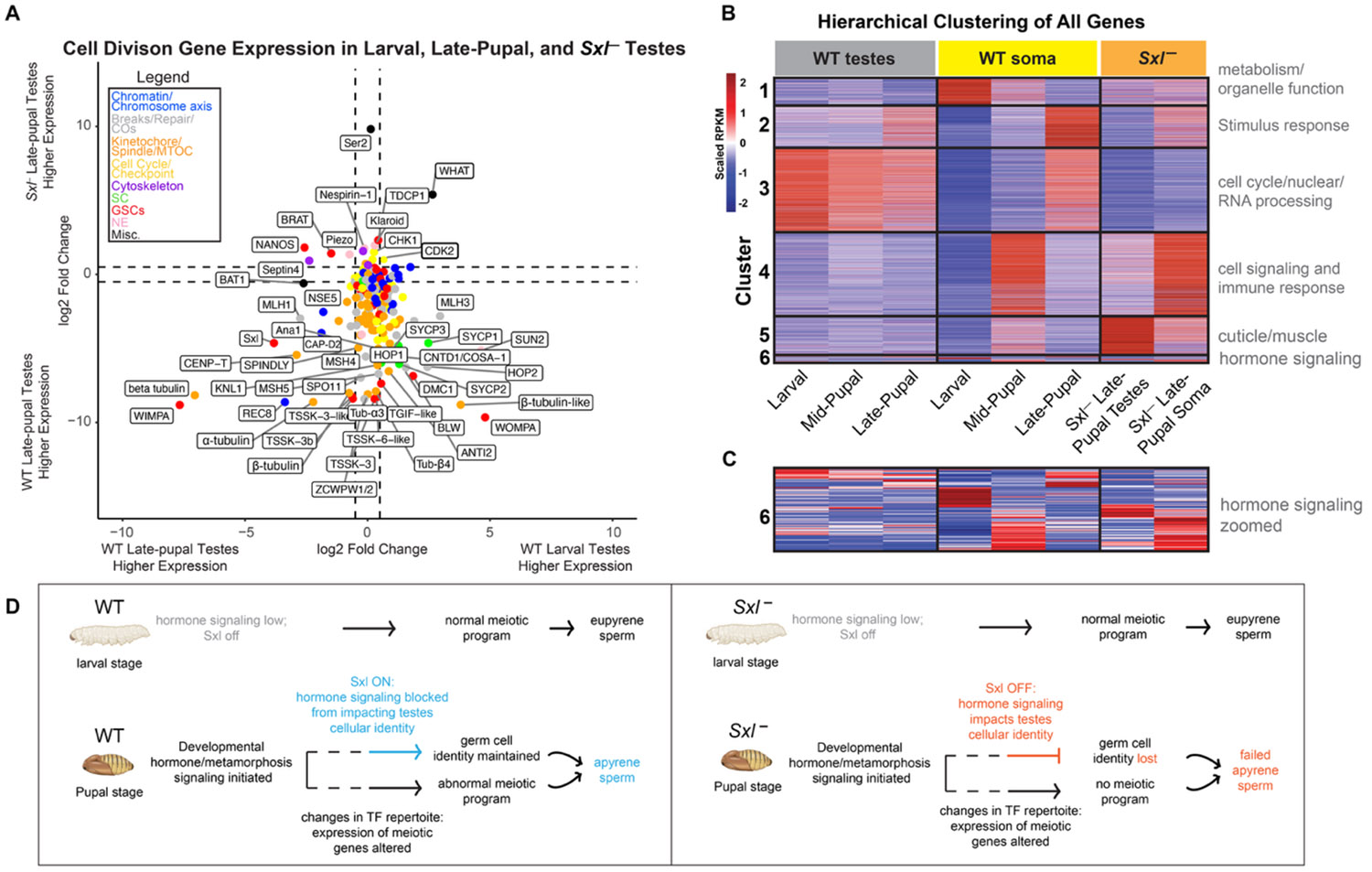
Cell division genes are suppressed independently of Sxl A. RNA-seq log2 fold change values of cell division genes between WT larval and late-pupal testes (X-axis) and *Sxl*^*−*^ and WT late-pupal testes (Y -axis). Select genes and their cell cycle roles [chromatin/chromosome axis (blue), breaks/repair/COs (gray), kinetochore/spindle/MTOC (orange), cell cycle/checkpoint (yellow), cytoskeleton (purple), synaptonemal complex (SC, green), germline stem cells (GSCs, red), nuclear envelope (NE, pink), and miscellaneous (black)] are indicated in the legend. B. Hierarchical clustering analysis of all expressed genes (scaled RPKM values) for the indicated tissues and developmental timepoints. Six distinct clusters are indicated to the left of the heatmap. Significant representative GO terms for each of the six clusters are indicated to the right of the heatmap. C. Expanded view of cluster 6 from panel B. D. Model. Hormone signaling genes are differentially expressed between tissues and time points, and so are global gene expression profiles. We propose a model where differences in hormone signaling lead to differences in putative transcription factors, which regulate either the eupyrene or apyrene spermatogenic pathways independently of *Sex-lethal* (*Sxl*). Expression of *Sxl* is also temporally regulated, and our data suggest that without functional Sxl, the transcriptional profile in testes more closely resembles that of somatic tissue, suggesting a role for Sxl in maintaining germ cell identity. These two pathways cooperatively ensure that the eupyrene to apyrene switch occurs successfully.

To determine if *Sxl* is required for the gene expression changes we described above, we acquired previously published *Sxl* knockout *(Sxl^−^) B. mori* pupae (14). We dissected testes from these pupae and adults and confirmed the previously-published phenotype of normal eupyrene sperm but abnormal apyrene sperm (Fig S9B-C). We then performed RNA-seq on *Sxl*^*−*^ *B. mori* late-pupal testes and somatic tissue (Fig 5A-C, Fig S10). We first focused on the expression of the above described 214 cell division genes (Dataset S2), comparing late-pupal *Sxl*^*−*^ testes to WT late-pupal testes. We found that only 7% (14/214) of cell division genes were upregulated (or ‘rescued’) in *Sxl*^*−*^ testes compared to WT, while 21% (44/214) were unchanged, and 73% (156/214) were downregulated (Fig 5A-C, Dataset S2). Among these 73% of downregulated genes were *sycp1, sycp2*, and *sycp3*. This finding suggests that *Sxl* is likely not required for, or at least not solely responsible for, the repression of cell division genes in apyrene spermatocytes and instead, it might play a role in maintaining the minimal levels of cell division gene expression required for the apyrene meiotic program.

### Loss of *Sex-lethal* leads to a somatic-like gene expression program in testes

Since there was no evidence for a rescue of the expression of cell division genes in *Sxl*^*−*^ late-pupal testes, we looked into other gene expression changes that could help explain why these mutants are unable to make functional apyrene sperm. Globally, we identified 4,687 genes with a significant increase in expression and 5,629 genes with a significant decrease in expression in *Sxl*^*−*^ late-pupal testes compared to WT late-pupal testes (Dataset S6). While some of these changes could be due to differences in genetic background of the *B. mori* strains being analyzed, it is also possible that loss of *Sxl* has a dramatic effect on downstream gene expression.

To test this latter possibility, we performed a K-means hierarchical clustering analysis on all expressed genes in all of our RNA-seq datasets (Dataset S6), revealing six distinct gene expression clusters amongst all samples. Interestingly, the global *Sxl*^*−*^ testes expression profile did not resemble any WT testes stage and instead was most similar to mid-pupal somatic tissue (Fig 5B). This was also true for the *Sxl*^*−*^ late-pupal somatic tissues. This observation was confirmed by principal component analysis, which is a mathematical linear reduction of data into 2-dimensional space (Fig S10). We also measured the Euclidean distance of all our replicates, a pairwise distance measurement, and found that the samples with the smallest average Euclidean distance to both *Sxl*^*−*^ late-pupal testes and somatic tissues were WT mid-pupal somatic tissues (Fig S10). Overall, this indicates that *Sxl*^*−*^ testes are more similar in gene expression to WT mid-pupal somatic tissues than they are to other testes tissues, and that *Sxl*^*−*^ late-pupal somatic tissues show developmentally delayed gene expression profiles. Thus, the tissue identity of *Sxl*^*−*^ testes and somatic tissues appear to be highly perturbed.

Since we found that *Sxl*^*−*^ late-pupal testes showed such a wildly different expression profile compared to WT late-pupal testes, we wanted to determine the types of genes that are dysregulated in *Sxl*^*−*^ testes within our clustering analysis. We therefore performed GO term analysis on each gene expression cluster (Fig 5B-C, Dataset S6). Cluster 5 genes showed increased expression in both *Sxl*^*−*^ late-pupal testes (7th column) and mid-pupal somatic tissues (5th column) and were most significantly associated with the GO term ‘structural constituent of cuticle’. In fact, we found that of the 219 annotated cuticle proteins in *B. mori*, 81% were increased in *Sxl*^*−*^ late-pupal testes compared to WT late-pupal testes (7th column compared to 3rd column). As cuticle genes are somatic in nature, their increased expression in *Sxl* testes is further support for the similarity in WT somatic and *Sxl*^*−*^ testes expression profiles noted above. Another interesting cluster was Cluster 2, which has high expression in WT late-pupal testes and late-pupal soma , but is decreased specifically in *Sxl*^*−*^ testes. This suggests that Cluster 2 genes are dependent on *Sxl* activity only in the testes. The most significant GO terms associated with this cluster were related to stimulus response, something else typically associated with somatic cell function (Dataset S7). Together, these findings support a model where *Sxl*^*−*^ late-pupal testes inappropriately express genes that are characteristic of somatic tissues and fail to express genes that are consistent with testes tissues, which may explain their inability to carry out the apyrene meiotic program.

To understand the basis of these differences in gene expression programs, we looked at the expression of transcriptional regulators. We identified putative transcription factors (pTFs) in the *B. mori* genome using a deep learning model specifically for the identification of transcription factors (DeepReg) (47). Out of the 13,437 annotated protein coding genes in the *B. mori* genome, DeepReg identified 2,141 genes as pTFs (Dataset S8), of which, 2,131 were found to be expressed in the testes based on our RNA-seq. We found that the repertoire of pTFs is significantly different between WT larval and pupal testes, which may contribute to the changes in gene expression we observed between these tissues (Fig S11). For example, compared to larval testes, 22% of pTFs are significantly downregulated in late-pupal testes, and 25% of pTFs become upregulated in pupal testes. In agreement with *Sxl*^*−*^ pupal testes failing to accurately carry out *any* kind of meiotic program, 68% of pTFs are differentially expressed between *Sxl*^*−*^ and eupyrene larval testes, and 65% of pTFs are differentially expressed between *Sxl*^*−*^ and late-pupal apyrene testes (Fig S11).

### Hormone signaling is perturbed in the absence of *Sex-lethal*

Since we didn’t see significant evidence that *Sxl* globally regulates the programmed transcriptional switch between eupyrene and apyrene meiosis, we sought to identify another factor that might be the apyrene spermatogenesis inducing factor (ASIF) (11, 12). Outside of *Sxl*, there is evidence that hormonal signaling plays a role in regulating the onset of meiosis in eupyrene spermatocytes and the switch between the eupyrene and apyrene programs (reviewed in (3)). However, the precise hormonal signaling pathway that would promote apyrene spermatogenesis is unclear, and there are likely differences between Lepidopteran species (48-50).

Using our RNA-seq data, we looked for changes in hormone-related gene expression that could support the identity of the ASIF in *B. mori*. We defined hormone signaling genes based on current NCBI annotations and specifically focused on genes annotated as part of ‘juvenile’ and ‘ecdysone’ signaling pathways since there is pre-existing evidence that these genes are involved in lepidopteran development and the eupyrene to apyrene switch (3). We also included additional annotated hormone signaling genes (e.g. broad-complex, nuclear receptor E74/E75, and hormone receptor 3) that have strong evidence of being downstream of juvenile and ecdysone hormone signaling in other insects such as *Drosophila* (51-55). Collectively, we refer this gene set as “hormone signaling genes”. Across developmental timepoints, only a minority of hormone signaling genes showed similar expression changes in both testes and somatic tissues (Fig 5B-C, S12, Dataset S1). Specifically, 22% (19/85) exhibited concordant differential expression between tissues at the same stage, whereas 59% (50/85) showed significantly different, tissue-specific responses. Thus, most hormone signaling genes display persistent testes- or somatic-biased expression rather than coordinated developmental regulation across tissues.

When comparing our *Sxl*^*−*^ late-pupal testes data to the WT late-pupal testes data, we found that 54% of hormone signaling genes (46/85) are differentially expressed (Fig 5B-C, S12, Dataset S1). For example, the annotated ecdysone-related genes *Cyp314a1* (52) and *Ecr* show a dramatic increase in expression in *Sxl*^*−*^ late-pupal testes compared to WT. Similarly, the *nuclear hormone receptor E75* and its co-factor *hormone receptor 3* (*Hr3*) (53, 54), are significantly increased in *Sxl*^*−*^ testes. Conversely, the ecdysone-related *broad-complex* (*Br-c*) (55), is decreased in expression in *Sxl*^*−*^ testes. Altogether, these results suggest that hormone signaling is perturbed in *Sxl*^*−*^ testes compared to WT late-pupal testes and could partially explain the inability to execute apyrene spermatogenesis.

Overall, our data suggest a mechanism where hormone signaling that is initiated during metamorphosis likely triggers multiple changes in the transcriptional programs of testes, including changes in transcription machinery, like pTFs.

## Discussion

The production of dimorphic sperm in Lepidoptera has remained enigmatic since its discovery in 1902 (1). Here, we identified multiple meiotic pathways that are transcriptionally altered during apyrene spermatogenesis, and these changes in expression correlate with changes in TF abundance. The fact that apyrene sperm morphs are essential for fertility (2, 10) suggests that apyrene meiosis is transcriptionally programmed to be doomed from the start. This model is in agreement with data from the wax moth, *Galleria mellonella*, which has distinct transcriptional programs between apyrene and eupyrene meiosis (30), and the gypsy moth, *Lymantria dispar*, which has meiotic errors as early as apyrene meiotic prophase I (24).

Our cytological and sequencing-based analyses indicate that during apyrene meiotic entry, chromosomes fail to properly form chromosome axes, possibly due to excess REC8-cohesin and Condensin I, and telomeres fail to localize to the nuclear edge, possibly due to transcriptional misregulation of SUN2 . Homologs fail to synapse, likely due to loss of SYCP1 and reduced SYCP2 and SYCP3. Expression of several checkpoint genes are reduced, possibly allowing for a quick progression through prophase despite these errors in pairing and synapsis (56, 57). Finally, all of these errors culminate in defective chromosome segregation.

Our observation of defects at the earliest stages of meiotic prophase in pupal testes could suggest that failed meiotic entry plays a crucial role in apyrene sperm formation. This would likely mean defects in the differentiation of germline stem cells (GSCs). Supporting this idea, we see that *brat*, which regulates asymmetric cell divisions in flies, including in GSCs (58), is significantly downregulated in apyrene meiosis (Dataset S1). However, other GSC genes, including many *nanos* orthologs and *pumilio*, are not differentially expressed between our apyrene and eupyrene data sets (Dataset S1), suggesting that GSCs are at least partially functional in pupal testes. Another possibility to explain the early loss of canonical meiotic progression in pupal testes is that chromosomes remain hyper-condensed in apyrene meiosis. This hyper-compaction could leave the genome in a more heterochromatic-like state where the normal expression of meiotic and cell cycle genes isn’t feasible.

Although Sxl is required for apyrene sperm formation (10, 14), our RNA-seq data suggest it does not broadly downregulate cell division genes during pupation. Imaging of *Sxl*^*−*^ apyrene sperm bundles shows defective nuclear positioning, consistent with a transitional eupyrene- to- apyrene state (10, 14), indicating that failed apyrene meiosis is unlikely due to increased meiotic gene expression. Instead, Sxl may act through a separate pathway independent of meiotic gene regulation. In *D. melanogaster*, Sxl is required to maintain female germ cell identity, and its loss leads to tumor formation and male-biased gene expression (59-61). While we have no evidence that Sxl regulates RNA splicing or male sexual identity in *B. mori*, our RNA-seq data indicate that *Sxl*^*−*^ testes fail to maintain WT apyrene or eupyrene testes expression profiles. One possible role of Sxl is therefore maintenance of germ cell identity in the testes; loss of this identity could prevent completion of apyrene spermatogenesis. Consistent with this model, disruption of germ cell identity via mutation of *prmt5* or *vasa* produces spermatogenic defects in *B. mori* that closely resemble those of Sxl mutants, including partial nuclear attrition and tail-mediated extrusion (9, 14, 29). A remaining caveat is that it is currently unknown whether Sxl functions in germ or somatic cells of the testes.

Notably, several cell division genes are upregulated rather than downregulated in *Sxl*^*−*^ testes, including cell cycle regulators and putative checkpoint proteins. These include CDK2, a key cell cycle regulator with meiosis-specific roles in prophase I telomere–nuclear envelope tethering and crossover regulation (62-64), and a second annotated CENP-E ortholog (LOC101739319; Dataset S2). Like the other annotated CENP-E, this protein lacks motor and microtubule-binding domains and instead contains a WH2 actin-binding region and C-terminal coiled-coil domains and is otherwise predicted to be largely disordered (Fig S13). Its closest ortholog is *D. melanogaster* SALS [*sarcomere length short*, a tropomysin-like protein that regulates actin filament polymerization (65-66; Fig S13; Dataset S5)], and we thus refer this gene as *what* (WH2 And Tropomyosin-like ). Because actin dynamics are critical for meiotic telomere clustering, spindle morphology, and chromosome segregation (67-70), misregulation of CDK2 and WHAT could underlie the telomere clustering and pairing defects observed in WT apyrene spermatocytes. Consistent with roles in canonical meiosis, both genes are more highly expressed in larval ovaries than pupal testes (Fig S14). Persistent expression of CDK2 and WHAT in *Sxl^−^* testes may therefore prevent apyrene spermatocytes from fully transitioning away from a canonical meiotic program, suggesting that Sxl promotes apyrene meiosis by bypassing CDK2-dependent checkpoints (71, 72) and actin-mediated surveillance via WHAT (73).

Our RNA-seq data reveals that hormone signaling genes show tissue-specific expression throughout development and each tissue likely has a complex response to systemic hormone signaling carried throughout the hemolymph. The significance of these differences is still unclear. *Sxl*^*−*^ testes show a perturbed hormonal gene expression response compared to their WT counterparts. Since there is previous evidence that a systemic hormone factor is involved in promoting the eupyrene-apyrene switch (11, 12) and *Sxl*^*−*^ testes show a perturbed hormonal signaling response, an attractive model for the role of *Sxl* is to integrate the systemic hormonal signaling response specifically within the testes, allowing for the transition from eupyrene to apyrene spermatogenesis. Our RNA-seq data indicate that without functional *Sxl* in pupal testes, there is either a loss of tissue identity or an inability to appropriately respond to hormonal signaling. Either way, the outcome on apyrene spermatogenesis without *Sxl* would likely be the inability to properly execute the apyrene spermatogenic program, consistent with the intermediate phenotype that has been previously described (10, 14).

Late-pupal somatic tissues show the same Sxl upregulation observed in pupal testes, resulting in similar gene expression profiles. *Sxl*^*−*^ late-pupal somatic tissues display perturbed expression and most closely resemble WT mid-pupal tissues. Although it remains unclear whether these changes are directly caused by Sxl, they are consistent with a role for Sxl in systemic hormonal signaling during development. Notably, Sxl mutants still initiate meiosis, likely because GSCs are already programmed to do so. Consistent with this, we detected no differential expression of GSC-associated genes between WT and Sxl mutant pupal testes, indicating intact stem cell identity. Instead, the altered hormonal environment may disrupt apyrene meiotic progression after initiation.

Beyond differences between apyrene and eupyrene spermatogenesis, our cytological analyses revealed that lateral element (LE) components of the SC are retained at metaphase I during *B. mori* male meiosis. In females, a large modified synaptonemal complex persisting through metaphase , termed the bivalent bridge , was previously described and shown to be derived from expanded LE proteins (34, 35, 74). Because *B. mori* females lack recombination, the bivalent bridge is proposed to ensure proper chromosome segregation at metaphase I by conjoining homologs into bivalents. This represents a repurposing of SC components for chromosome segregation. Our finding that LE proteins also retained in male meiosis suggests a similar repurposing of SC components ; however, because males undergo recombination (75, 76), retained LEs may serve an additional or backup role in homolog linkage when crossovers fail.

In addition to characterizing conserved cell division genes in eupyrene and apyrene meioses, we identified new *B. mori* orthologs of conserved meiotic proteins and two novel factors: WHAT and BAT1. WHAT is a putative actin-binding protein downregulated during apyrene meiosis and may regulate chromosome movement and segregation; its reduction could underlie telomere tethering and spindle defects observed previously (3, 22, 77). BAT1 is highly expressed exclusively during apyrene meiosis and, despite being insect-specific, contains domains consistent with a role in flagellar motor activity, suggesting a function in apyrene sperm motility (3, 10, 78).

Overall, our data suggest that hormone signaling during metamorphosis drives widespread remodeling of transcriptional programs in the testes, including changes to transcription-related factors like pTFs. We propose a model where these changes in pTF profiles alter the expression of cell division genes in the testes. At the same time, *Sxl* is turned on, and this blocks hormone signaling from globally impacting testes cellular identity. The combination of these two things leads to testes that produce apyrene sperm. Without *Sxl*, developmental hormones can alter the tissue identity of testes, leading to pupal testes assuming a more somatic-like tissue fate that fail to properly execute the apyrene meiotic program (Fig 5D).

In summary, by combining cytological and sequencing-based methods, we find that differences in the dichotomous spermatogenic pathways in *B. mori* initiate early in meiotic prophase I, and our findings reveal significant insights into two converging molecular pathways that promote the formation of the two sperm morphs in Lepidoptera.

## Materials and Methods

For complete Materials and Methods, please see Appendix S1.

### Silkworms:

Silkworms were obtained from Educational Science https://www.educationalscience.com, FramsChams Chameleon Breeders (https://framschams.com/collections/silkworms), or the National Bio-Resource Project (NBRP) of the MEXT, Japan at Kyushu University in Fukuoka, Japan (https://shigen.nig.ac.jp/silkwormbase/about_kaiko.jsp). For staging, see Appendix S1.

### Imaging:

IF and IF/FISH were performed based on previously described methods (17, 38, 79). For details on antibodies, slide preparation, imaging, and quantification, please see Appendix S1.

### RNA-sequencing:

Total RNA was isolated from tissues using the Direct-zol RNA Miniprep Plus Kit (Zymo Research, Tustin, CA) following manufacturer’s instructions. rRNA-depleted libraries were made using the Zymo-Seq RiboFree Total RNA Library Kit (Zymo Research) and indexed with Zymo-Seq Unique Dual Index (UDI) Primer Plate (Zymo Research) according to the manufacturer’s instructions. Libraries were sequenced on a NovaSeq 6000 SP200 flow cell. For RNA-seq analysis, see Appendix S1.

### ATAC-sequencing:

ATAC-seq library generation was performed using the ATAC-seq Kit (Active Motif, catalog 53150). See Appendix S1 for details and for ATAC-seq analysis.

## Supplementary Material

Supplemental Materials

Dataset S9

Dataset S7

Dataset S6

Dataset S2

Dataset S4

Dataset S8

Dataset S5

Dataset S3

Dataset S1

## Data Availability

Genomics sequencing data have been deposited in SRA (SRR34018377-SRR34018405). Stowers Institute original data underlying this manuscript can be accessed from the Stowers Original Data Repository at https://www.stowers.org/research/publications/LIBPB-2548. Other data are included in the article and/or supporting information.

## Significance

Male moths and butterflies produce two different sperm types: one with DNA that fertilizes eggs (eupyrene), and one without DNA (apyrene), which facilitates fertilization. We use microscopy and RNA sequencing to study how these two sperm morphs are made. We find that apyrene-destined cells fail to accurately perform critical steps of early meiosis due to misregulation of conserved cell division genes. These changes in gene expression are likely regulated by developmental hormones and are independent of a known apyrene factor, *Sex-lethal*. These results shed light on a long-standing mystery in the field of chromosome biology: how sperm without DNA are made and how the same animal can switch from making normal to DNA-less sperm.
