# Supplementary material for: Programmed meiotic errors facilitate dichotomous sperm production in the silkworm, Bombyx mori
Source: Proc Natl Acad Sci U S A. Author manuscript; Available in PMC 2026 Apr 6. (PMC12956816; doi:10.1073/pnas.2520991123)
Supplement: Supplemental Materials [file NIHMS2157370-supplement-Supplemental_Materials.pdf]

Supplemental Figures and Legends

Supplementary Figure 1. Telomere clustering and SC formation in eupyrene spermatogenesis in *B. mori*.

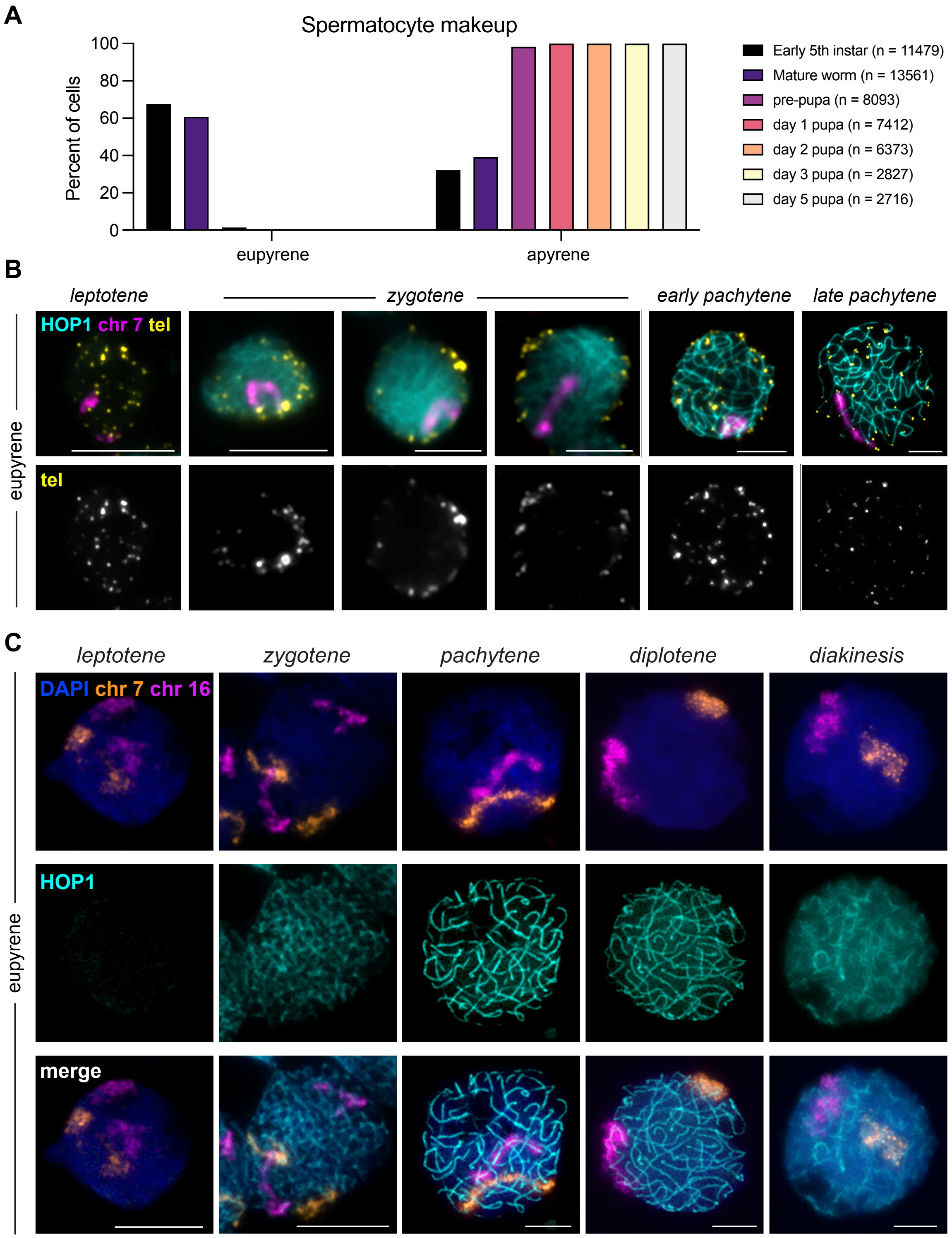

A. Timing of eupyrene and apyrene spermatogenesis during silkworm development. Eupyrene spermatogenesis occurs primarily in larval stages while apyrene development occurs in pupation. A mix of both eupyrene and apyrene production occurs from 5th instar to around pre-pupation.

B. Quantification of eupyrene and apyrene spermatocytes throughout development. Using SYCP2 to distinguish between eupyrene and apyrene spermatocytes, the ratio between eupyrene and apyrene spermatocytes as a percentage of each cell type was calculated for the stages 5th instar, mature instar, pre-pupa, day 1 pupa, day 2 pupa, day 3 pupa, and day 5 pupa. In 5th and mature instars, the majority of cells are eupyrene spermatocytes (68% in 5th instar and 61% in mature instars). In pre-pupa, the majority are apyrene spermatocytes (98%). At remaining stages, no eupyrene spermatocytes were found. 5th instar, n = 11479. Mature instars, n = 13561. Pre-pupa, n = 8093. Day 1 pupa, n = 7412. Day 2 pupa, n = 6373. Day 3 pupa, n = 2827. Day 5 pupa, n = 2716. Counting was done using a mechanical cell counter. All counting was done on prophase I cells. Data came from cryosectioned slides.

C. Representative cells from 5th instar larval testes (eupyrene meiosis) spreads showing HOP1 (lateral element; cyan), Oligopaints for chr 7 (magenta), and telomere repeat FISH probes (yellow). As homologs align at zygotene, telomeres concurrently cluster around the nuclear periphery before dispersing again at the end of pachytene. Scale bar is 5 μm.

D. Cells from 5th instar larval testes (eupyrene meiosis) chromosome spreads. Cells are labeled with HOP1 (cyan), Oligopaints for chr 7 (orange), and Oligoppaints for chr 16 (magenta). DAPI is shown in blue. HOP1 localization to chromosomes begins in early zygotene (second column) and remains chromosome-associated throughout prophase I. Scale bar is 5 μm.

Supplementary Figure 2. Apyrene spermatogenesis in *B. mori* has aberrant SC formation.

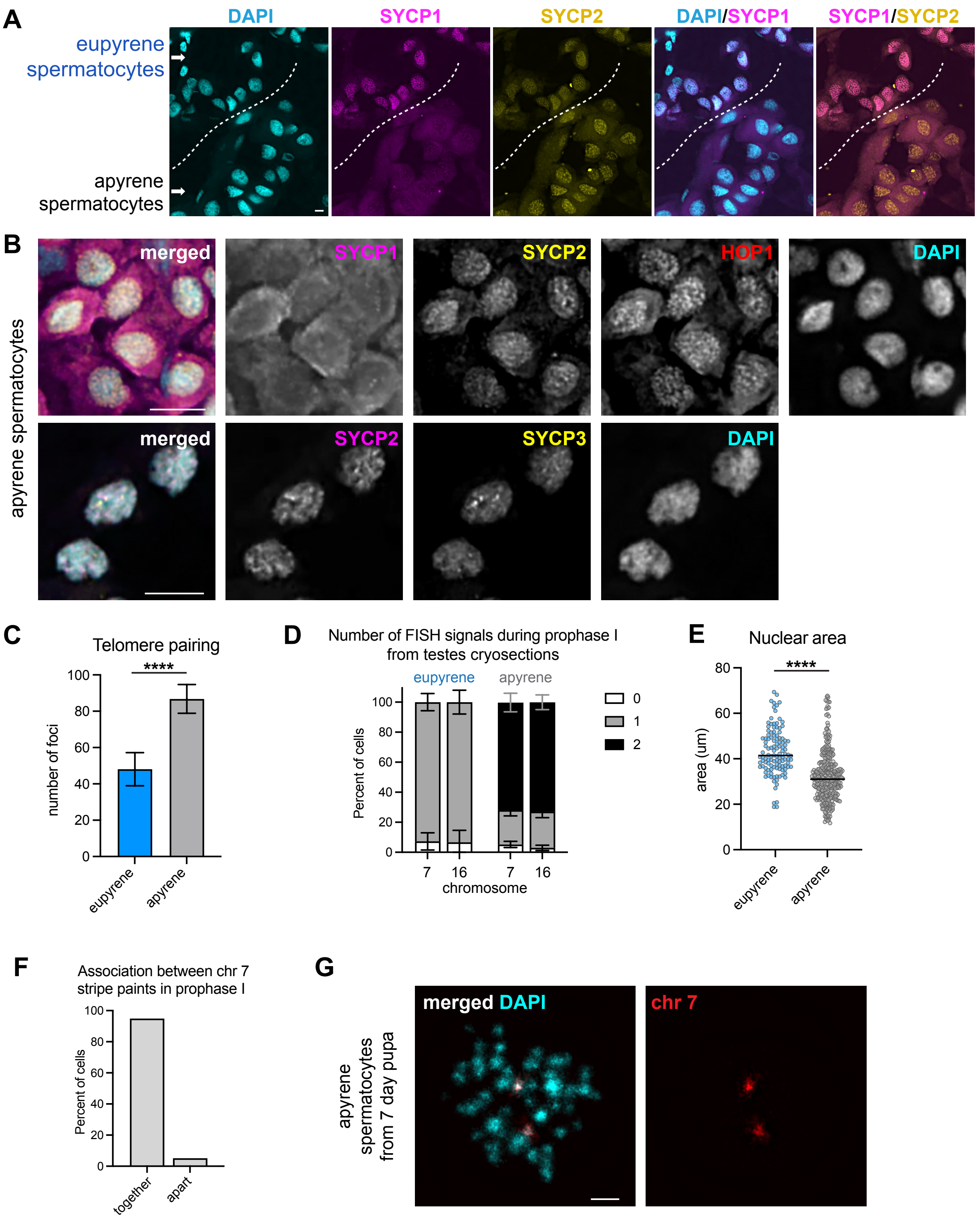

A. In mature larvae, both eupyrene and apyrene spermatocytes are detected. Apyrene spermatocytes show no SYCP1(magenta) and only short threads of SYCP2 (yellow). Eupyrene spermatocytes have full length threads of SYCP1(magenta) and SYCP2 (yellow). DAPI is shown in cyan. Scale bar is 5 µm. Images are maximum-intensity projections of the deconvolved *z*-series though the selected nuclei. *Associated with Figure 1.*

B. Representative images of apyrene spermatocytes from day 1 pupal testes. Top row: DAPI (cyan), SYCP1 (magenta), SYCP2 (yellow), and HOP1 (red). Bottom row: DAPI (cyan), SYCP2 (magenta), and SYCP3 (yellow). HOP1, SYCP2, and SYCP3 are localized to chromatin, but SYCP1 antibody shows only weak, non-specific signal. SYCP2, SYCP3, and HOP1 appear to form short threads. Scale bar is 5 µM. Images are maximum-intensity projections of the deconvolved *z*-series though the selected nuclei. *Associated with Figure 1.*

C. Bar graph showing number of telomeric FISH signals per nucleus in eupyrene (5th instar larvae) and apyrene (day 4 pupa) spermatocytes. Average number of telomeres was 48.1 ± 9.1 for eupyrene spermatocytes (n = 79) and 86.8 ± 7.9 for apyrene spermatocytes (n = 68). P<0.0001; were unpaired t-test (Kolmogorov-Smirnov test) between distributions.

D. Quantification of the number of whole chromosome Oligopaint signals in prophase I cells. Data from eupyrene spermatocytes from 5th and mature instars averaged together had 1 signal in 92.7% & 93.3% (chr 7 & chr 16) of all samples (n = 199). This indicates that eupyrene spermatocytes are paired, with the error coming from small deviations due to sectioning and counting. The percentage that had zero signal was 7.3% and 6.67% (chr 7 and chr 16, n = 199). None had 2 signals. Conversely, apyrene spermatocytes from pre-pupa, day 1 pupa, day 2 pupa, and day 3 pupa averaged together 2 signals in 71.6% & 72.8% (chr 7 & chr 16) of all samples (chr 7, n = 779, chr 16, n = 996). This indicates that these chromosomes are not paired. The percentage that had 1 signal was 22.9% & 24.2% (chr 7, n = 779, chr 16, n = 996). The percentage that had zero signal was 5.21% & 2.94% (chr 7, n = 779, chr 16, n = 996). The lack of signal is likely due to sectioning and counting through different z-sections. Error bars are standard deviation. Data came from sectioned slides.

E. Dot plot showing area of eupyrene nuclei (early 5th instar) and apyrene nuclei (day 1-3 pupae). The nuclei of eupyrene prophase I spermatocytes are significantly bigger than apyrene prophase I spermatocytes. DAPI was used as a proxy to measure nuclear size. Average size of eupyrene 5th instar nuclei was 43.13 µm (n = 116), which was statistically bigger than the average area of nuclei in apyrene spermatocytes (33.3 µm, n = 320), For area calculations, see materials and methods. P<0.0001; were unpaired t-test (Kolmogorov-Smirnov test) between distributions. Data came from sectioned slides.

F. Quantification of chromosome fragmentation as determined by the association of two sub-chromosomal “stripe” paints. Data shown are from day 1 pupae and demonstrate that 95% of all apyrene spermatocytes have their chr 7 s1 and chr 7 s2 associated, showing lack of fragmentation (n = 236).

G. Metaphase I spreads from day 7 pupal testes showing that apyrene spermatocytes at metaphase I do not have 28 bivalents as they would in eupyrene spermatocytes, DAPI (cyan) and whole chromosome Oligopaint for chr 7 (red). Scale bar is 5 μm. Images are maximum-intensity projections of the deconvolved *z*-series though the selected nuclei.

Supplementary Figure 3. SYCP2, HOP1, and cohesin component REC8 remain on metaphase I chromosomes in eupyrene meiosis.

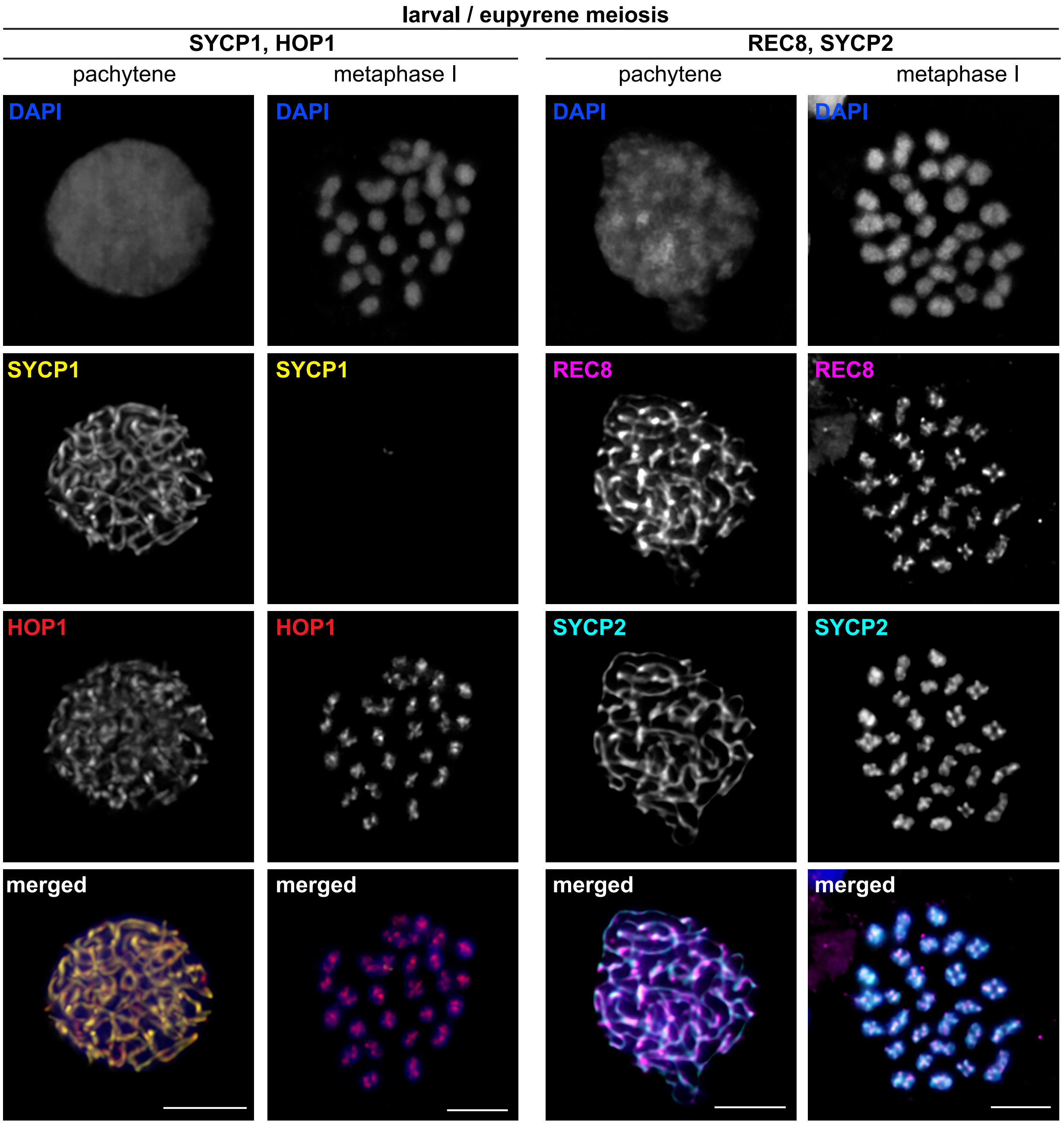

Representative images of IF on pachytene (left) and metaphase I (right) cells from larval testes chromosome spreads. Paired pachytene and metaphase I cells were in the same field of cells from the same slides. Left two columns: SYCP1 (yellow) and HOP1 (red). Right two columns: REC8 (magenta) and SYCP2 (cyan). HOP1, REC8, and SYCP2 remain on chromosomes through metaphase I in eupyrene meiosis. DAPI is shown in blue. Scale bar is 5 μm.

Supplementary Figure 4. SYCP2, HOP1, and cohesin component REC8 remain on metaphase I chromosomes in apyrene meiosis.

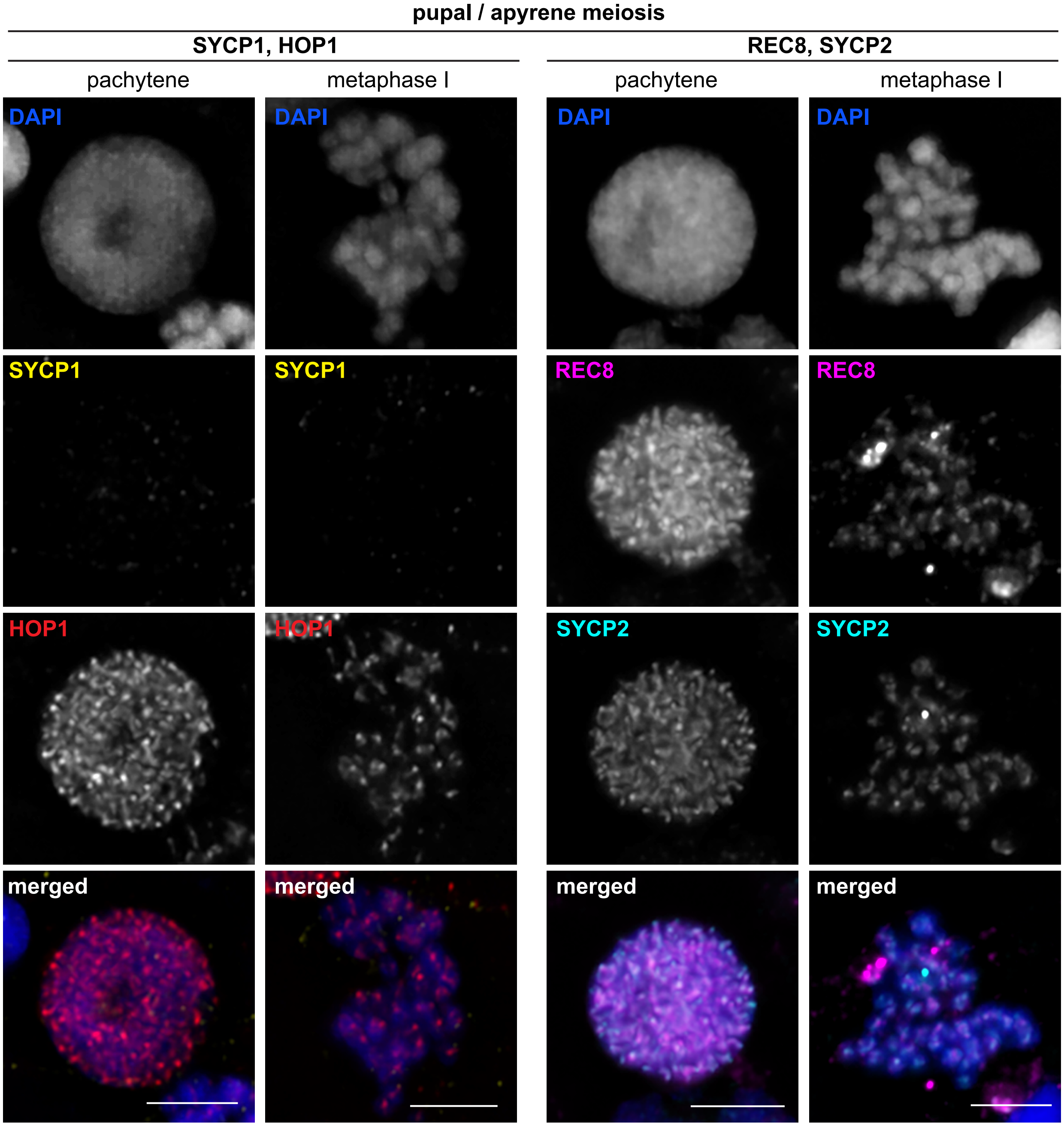

Representative images of IF on pachytene (left) and metaphase (right) cells from pupal testes chromosome spreads. Paired pachytene and metaphase cells were in the same field of cells from the same slides. Left two columns: SYCP1 (yellow) and HOP1 (red). Right two columns: REC8 (magenta) and SYCP2 (cyan). HOP1, REC8, and SYCP2 remain chromatin-associated through metaphase I in apyrene meiosis. DAPI is shown in blue. Scale bar is 5 μm.

Supplementary Figure 5. Normal meiosis in eupyrene spermatogenesis.

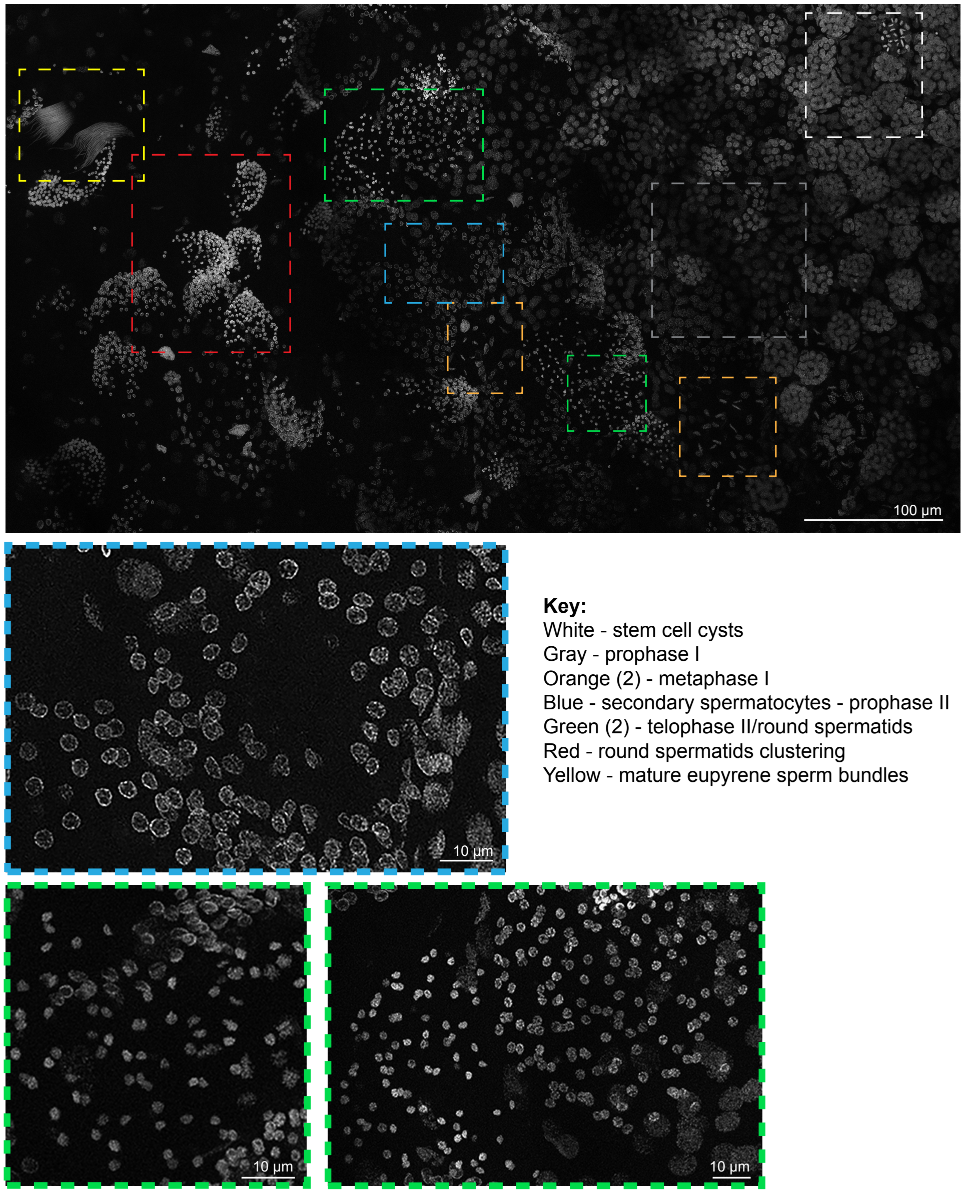

DAPI staining on whole-mount WT larval testis. White: Stem cell cysts. Gray: Cysts in prophase I. Orange: Cysts in metaphase I. Blue: Cysts of secondary spermatocytes in prophase II. Green: Group of cysts in secondary spermatocytes in telophase II and round spermatids. Red: Round spermatids clusters. Yellow: Mature eupyrene sperm bundles. Scale bar is 100 µm. Bottom: Zoomed in views of cysts of secondary spermatocytes in prophase II (blue) and telophase II (green). Scale bar is 10 µm.

Supplementary Figure 6. Cell division genes are misregulated during apyrene meiosis

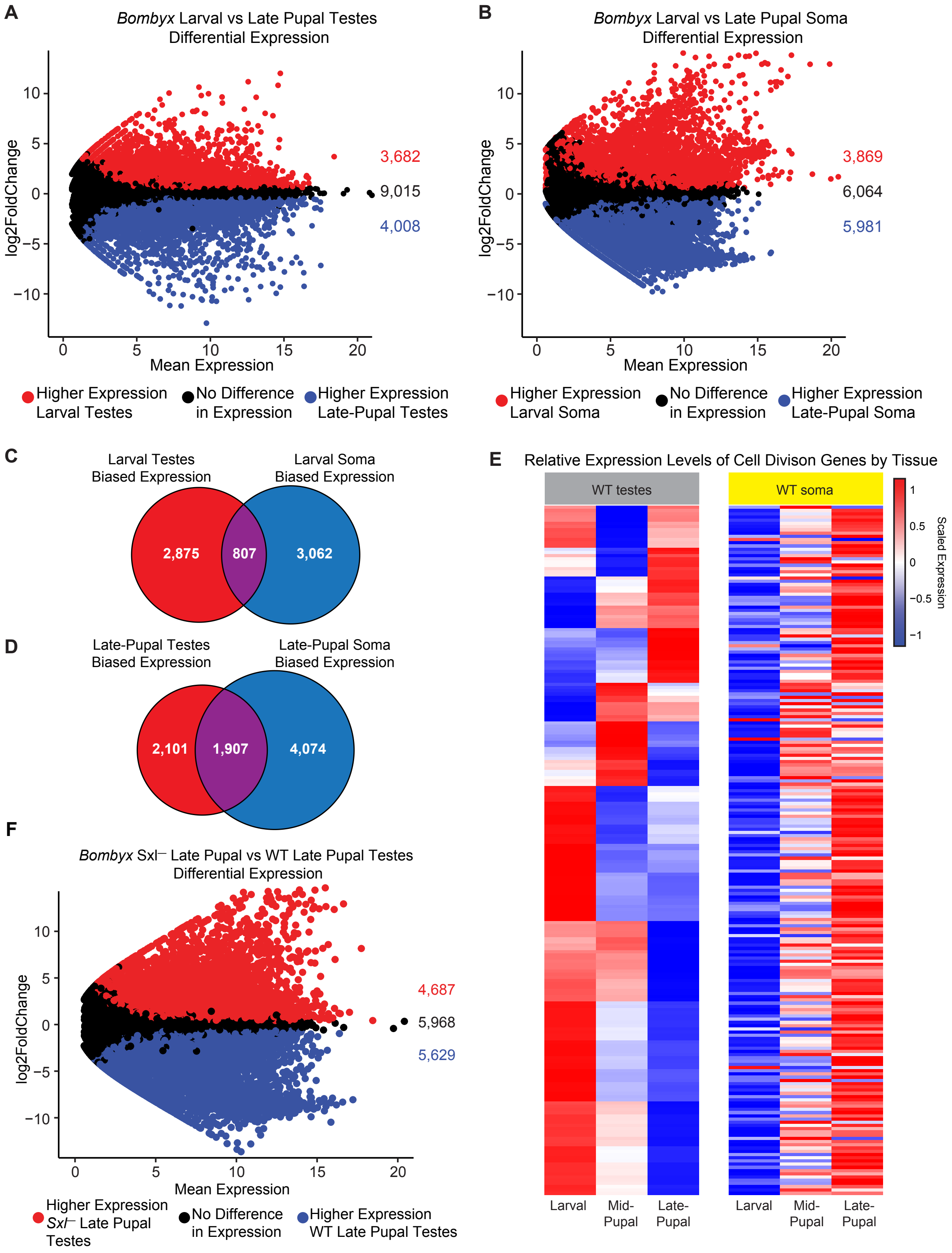

A. MA plot of WT larval vs late-pupal testes gene expression. Red dots indicate genes that are significantly higher expressed in WT larval testes, blue dots indicate genes that are significantly higher expressed in WT late-pupal testes, and black dots indicate genes that are not differentially expressed.

B. MA plot of WT larval vs late-pupal somatic gene expression. Red dots indicate genes that are significantly higher expressed in WT larval soma, blue dots indicate genes that are significantly higher expressed in WT late-pupal soma, and black dots indicate genes that are not differentially expressed.

C. Intersection of genes that are significantly higher expressed in larval testes vs late-pupal testes and larval soma vs late-pupal soma.

D. Intersection of genes that are significantly higher expressed in late-pupal testes vs larval testes and late-pupal soma vs larval soma.

E. Heatmap of cell division gene expression (scaled RPKM values) in testes and somatic tissues across development. Genes are hierarchically clustered by their expression in testes tissues.

F. MA plot of *Sxl*$^{-}$ and WT late-pupal testes. Red dots indicate genes that are significantly higher expressed in *Sxl*$^{-}$ late-pupal testes, blue dots indicate genes that are significantly higher expressed in WT late-pupal testes, and black dots indicate genes that are not differentially expressed.

Supplementary Figure 7. Cell division gene expression comparison in larval testes, larval ovaries, and late-pupal testes.

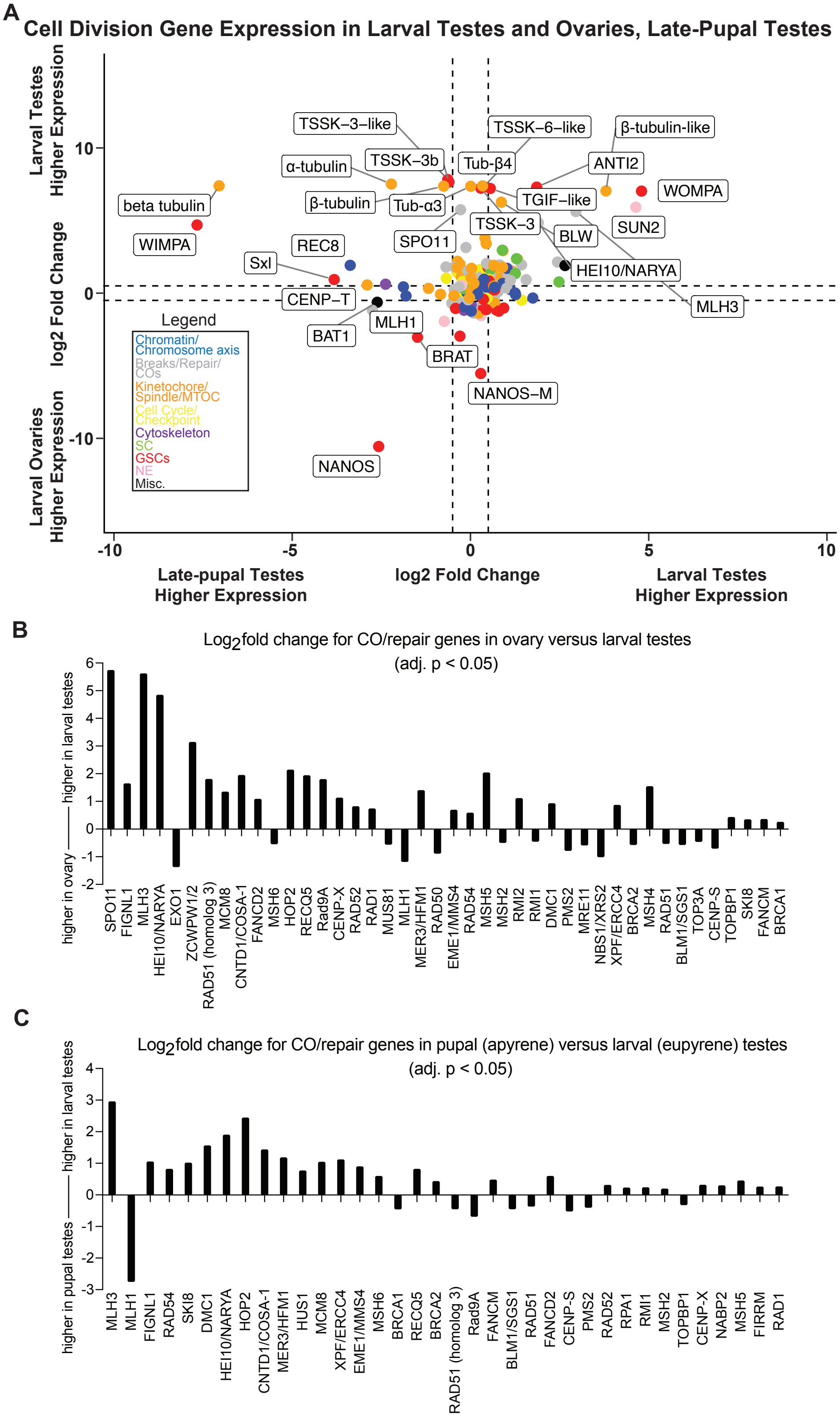

A. RNA-seq log2 fold change values of cell division genes between WT larval and late-pupal testes (X-axis) and WT larval testes and WT larval ovaries (Y-axis). Select genes and their cell cycle roles, chromatin/chromosome axis (blue), breaks/repair/COs (gray), kinetochore/spindle/MTOC (orange), cell cycle/checkpoint (yellow), cytoskeleton (purple), synaptonemal complex (SC, green), GSCs (germline stem cells, red), nuclear envelope (NE, pink) and miscellaneous (black) are indicated in the legend.

B. Bar graphs showing the log2 fold change (Y-axis) for CO/repair genes (X-axis) that are significantly differentially expressed (adj. $p < 0.005$) between larval testes and larval ovaries.

C. Bar graphs showing the log2 fold change (Y-axis) for CO/repair genes (X-axis) that are significantly differentially expressed (adj. $p < 0.005$) between larval testes and pupal testes.

Supplementary Figure 8. Chromatin is broadly more accessible by ATAC-seq in larval testes compared to pupal testes.

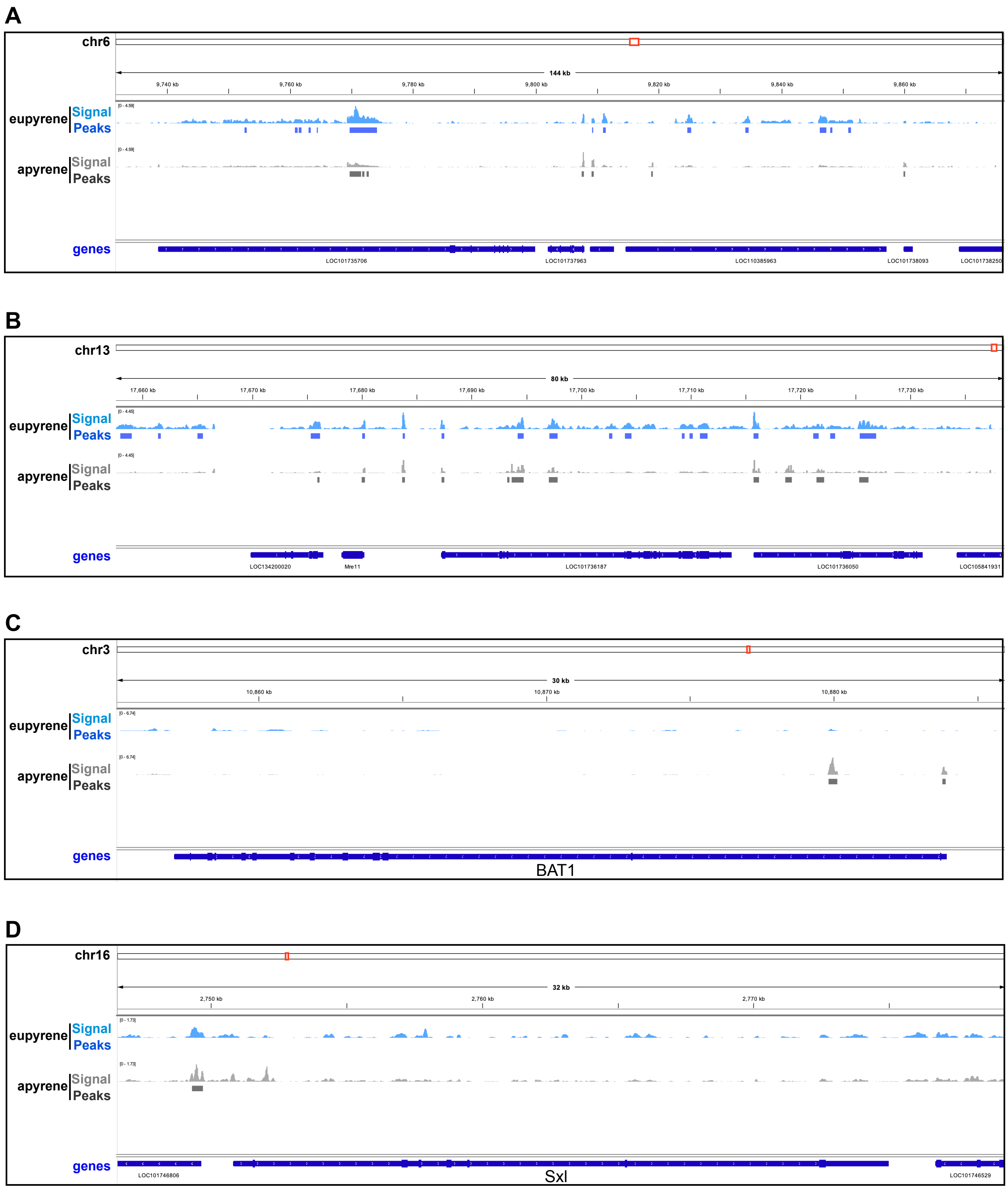

A-B. Representative screenshots of ATAC-seq tracks and peaks showing the global increase in accessibility in larval testes chromatin (eupyrene meiosis; blue) compared to pupal testes chromatin (apyrene meiosis; gray). The notable exception to this is increased accessibility at the promoters of genes which become upregulated during apyrene meiosis, such as BAT1 (C) and Sxl (D).

Supplementary Figure 9. *Sxl* gene expression tracks and sperm bundles.

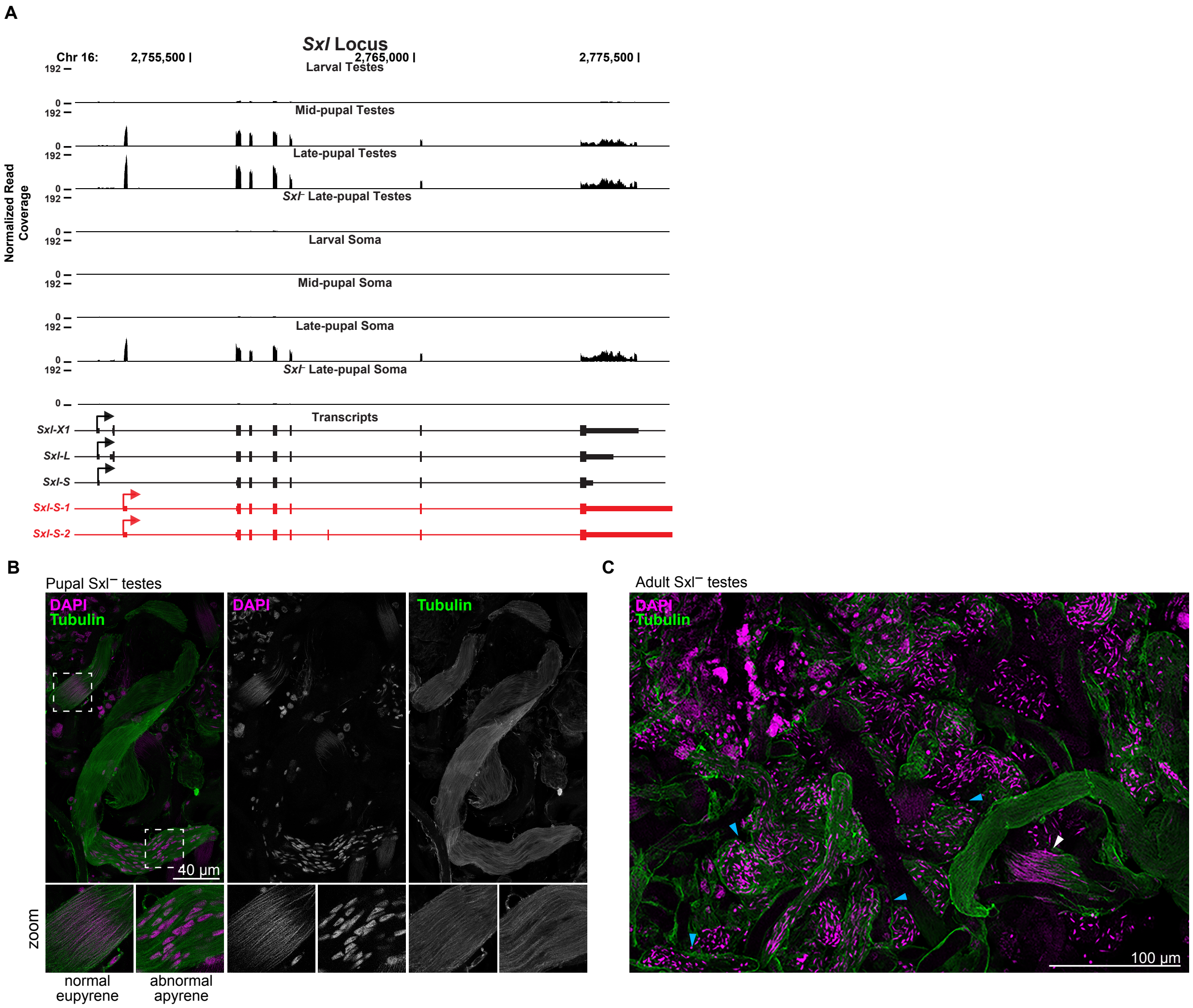

A. RNA-seq normalized read coverage tracks of the *B. mori Sxl* locus for the indicated tissues and developmental timepoints. Arrows indicate transcriptional start sites, small rectangles indicate untranslated regions, large rectangles indicate open reading frames. Black transcripts represent NCBI annotated transcripts and red transcripts indicate *de novo* assembled transcripts from our analysis.

B. Sperm bundles from *Sxl*$^{-}$ late-pupal testes. DAPI is shown in magenta and tubulin in green. Scale bar is 50 µm. Bottom: Zoomed in views of nuclei in eupyrene and apyrene sperm bundles.

C. Sperm bundles from *Sxl*$^{-}$ adult testes. DAPI is shown in magenta and tubulin in green. White arrowheads indicate normal eupyrene sperm bundles. Blue arrowheads indicate abnormal apyrene sperm bundles. Scale bar is 100 µm.

Supplementary Figure 10. RNA-seq hierarchical replicate clustering and PCA plots.

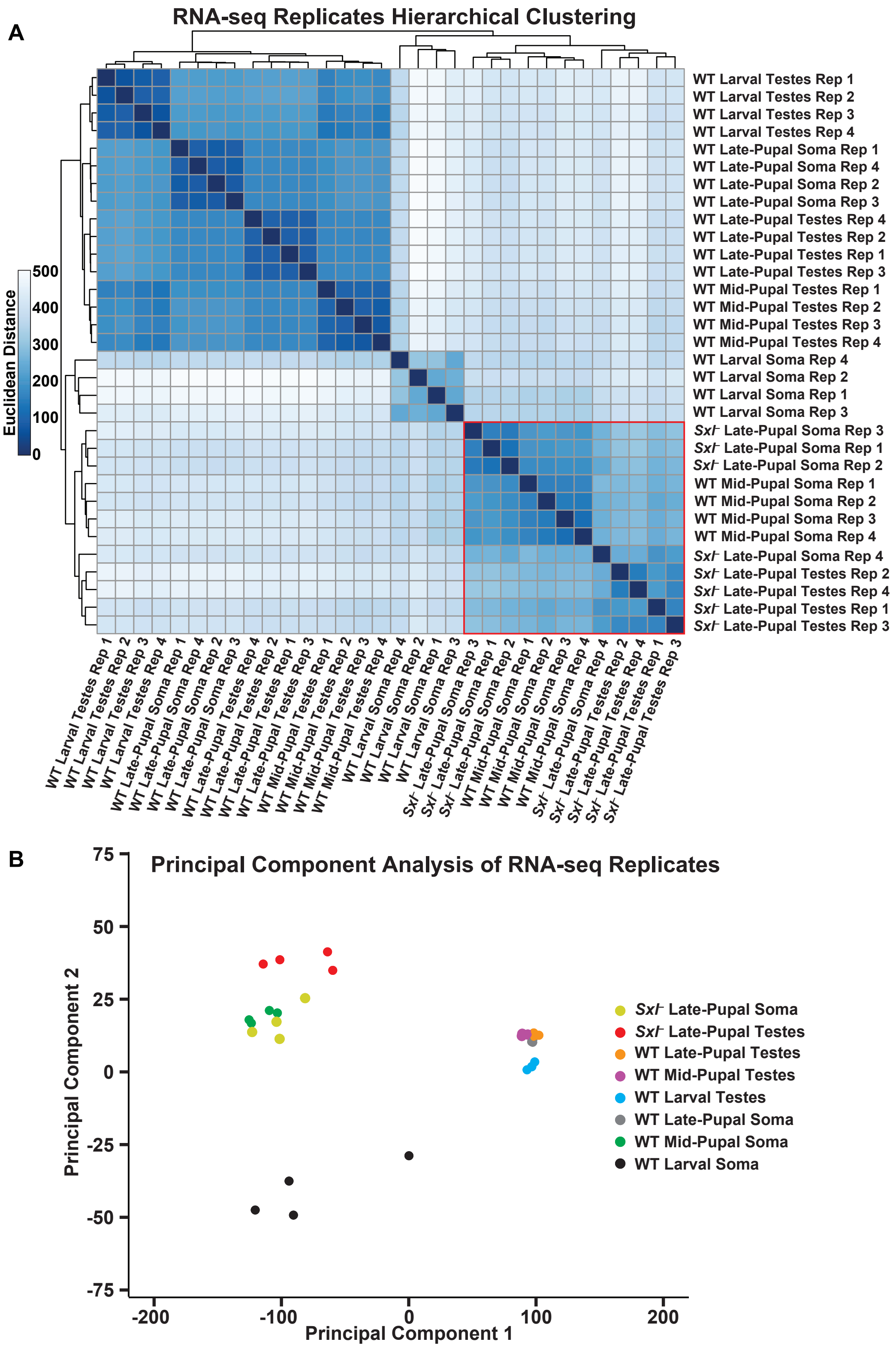

A. Heatmap measuring Euclidean distances of the respective RNA-seq replicates used in our analysis. Red box shows the location of the WT mid-pupal soma and *Sxl*⁻ late-pupal testes and soma samples.

B. Principal component analysis plot of *Sxl*⁻ late-pupa soma (yellow), *Sxl*⁻ late-pupa testes (red), WT late-pupal testes (orange), WT mid-pupal testes (purple), WT larval testes (blue), WT late-pupal soma (gray), WT mid-pupal soma (green), and WT larval soma (black) RNA-seq replicates used in our analysis.

Supplementary Figure 11. Putative transcription factor expression between WT late-pupal testes and WT larval testes and *Sxl*⁻ late-pupal testes.

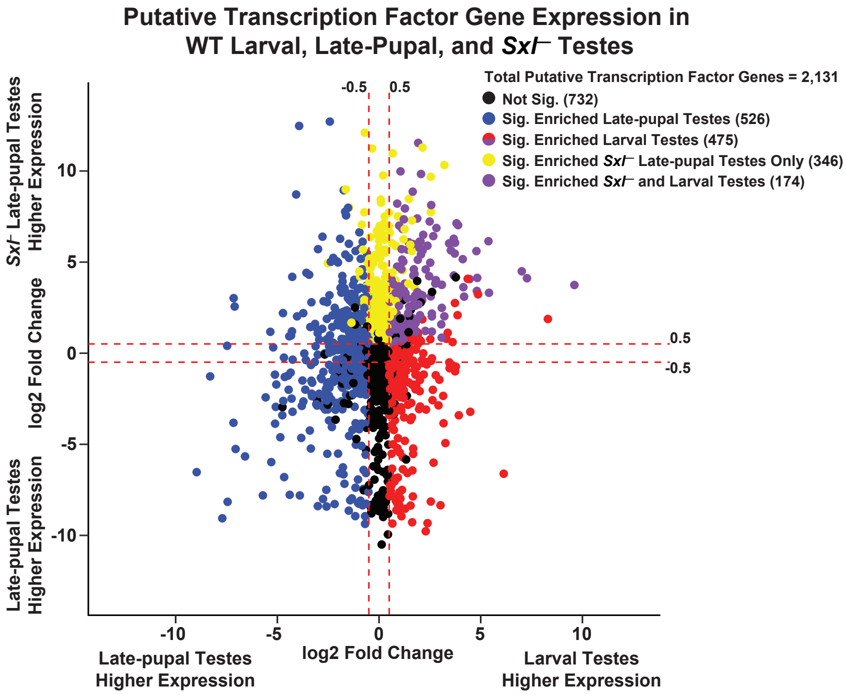

RNA-seq log2 fold change values of putative transcription factor genes between WT larval testes and WT late-pupal testes (X-axis) and *Sxl*⁻ late-pupal testes and WT late-pupal testes (Y-axis). Black dots indicate genes that are not differentially expressed. Blue dots indicate genes that are significantly higher expressed in WT late-pupal testes compared to WT larval testes. Red dots indicate genes that are significantly higher expressed in WT larval testes compared to WT late-pupal testes. Yellow dots indicate genes that are significantly higher expressed in *Sxl*⁻ late-pupal testes compared to WT late-pupal testes. Purple dots indicate genes that are significantly higher expressed in both WT larval testes and *Sxl*⁻ late-pupal testes compared to WT late-pupal testes.

Supplementary Figure 12. Relative gene expression heatmaps of annotated hormone signaling genes.

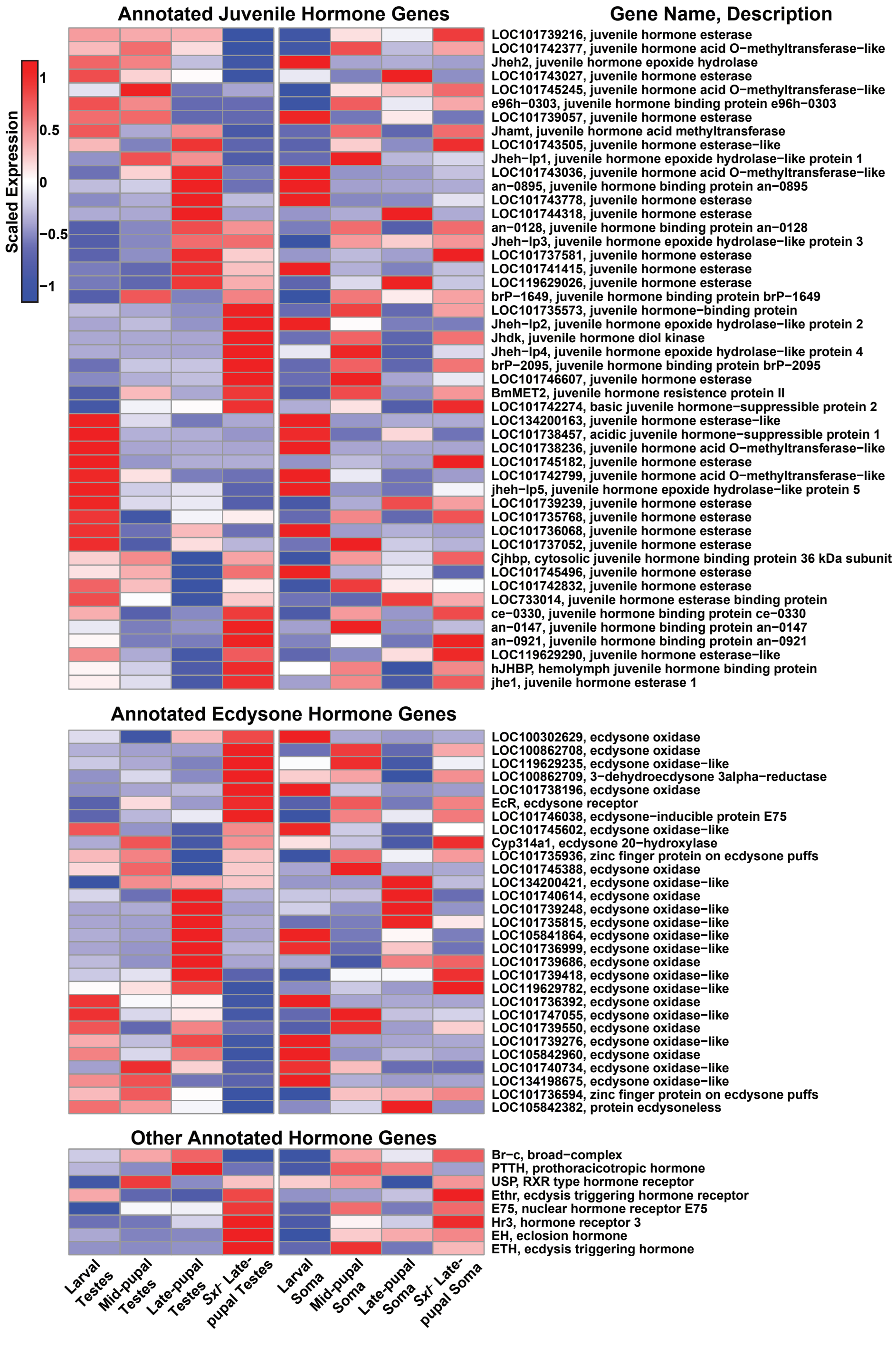

Heatmap of annotated hormone gene expression (scaled RPKM values) in testes and somatic tissues across development. Genes are hierarchically clustered by their expression in testes tissues.

Supplementary Figure 13. WHAT is a putative actin binding protein involved in *B. mori* eupyrene meiosis.

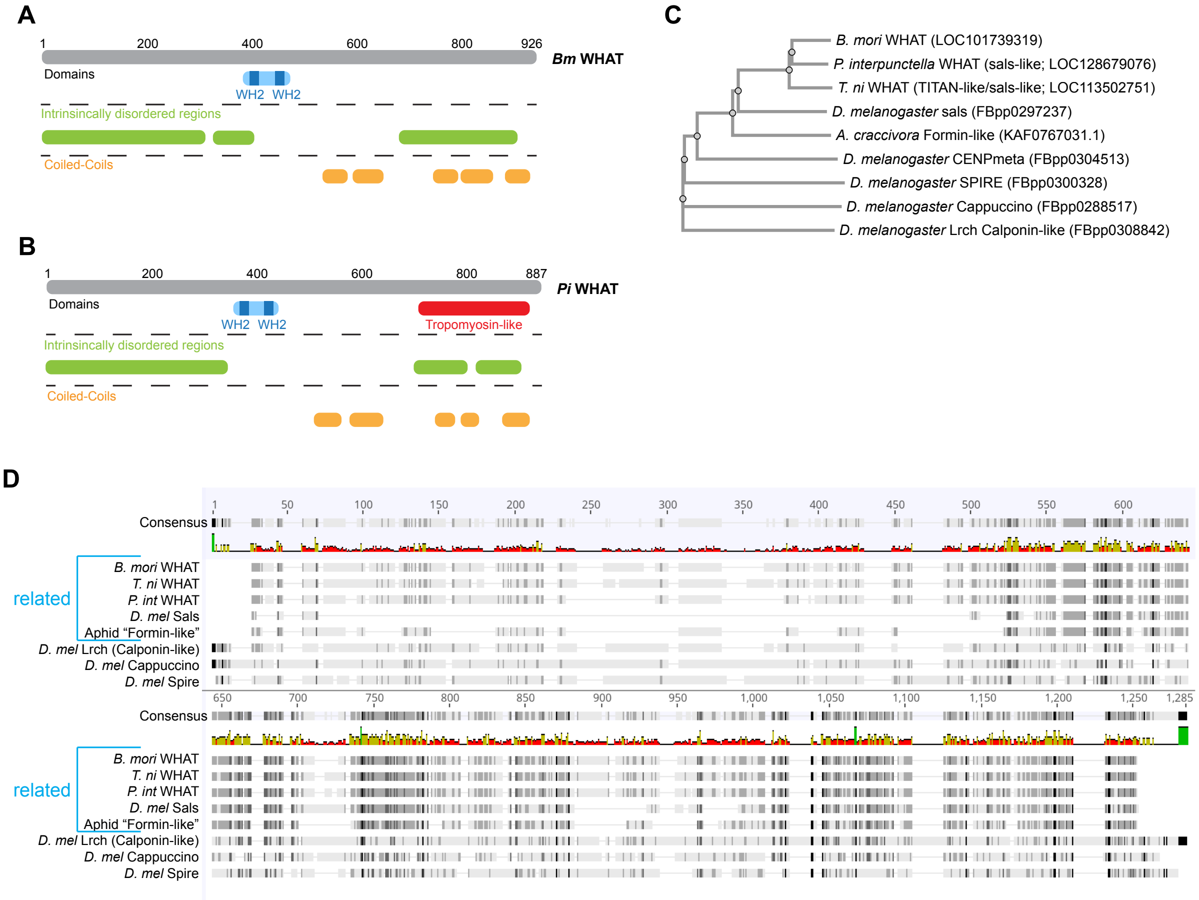

A. pfam protein domains identified in *B. mori* WHAT.

B. pfam protein domains identified in *P. interpunctella* WHAT.

C. Neighbor-joining tree of putative WHAT-like proteins in moths and flies, with one putative ortholog identified in aphids.

D. Multiple sequence alignment showing conservation, where darker color means more conserved. Based on the alignment, only the top 5 proteins are predicted to be orthologous.

Supplementary Figure 14. Cell division gene expression comparison between larval ovaries, larval testes, and late-pupal testes.

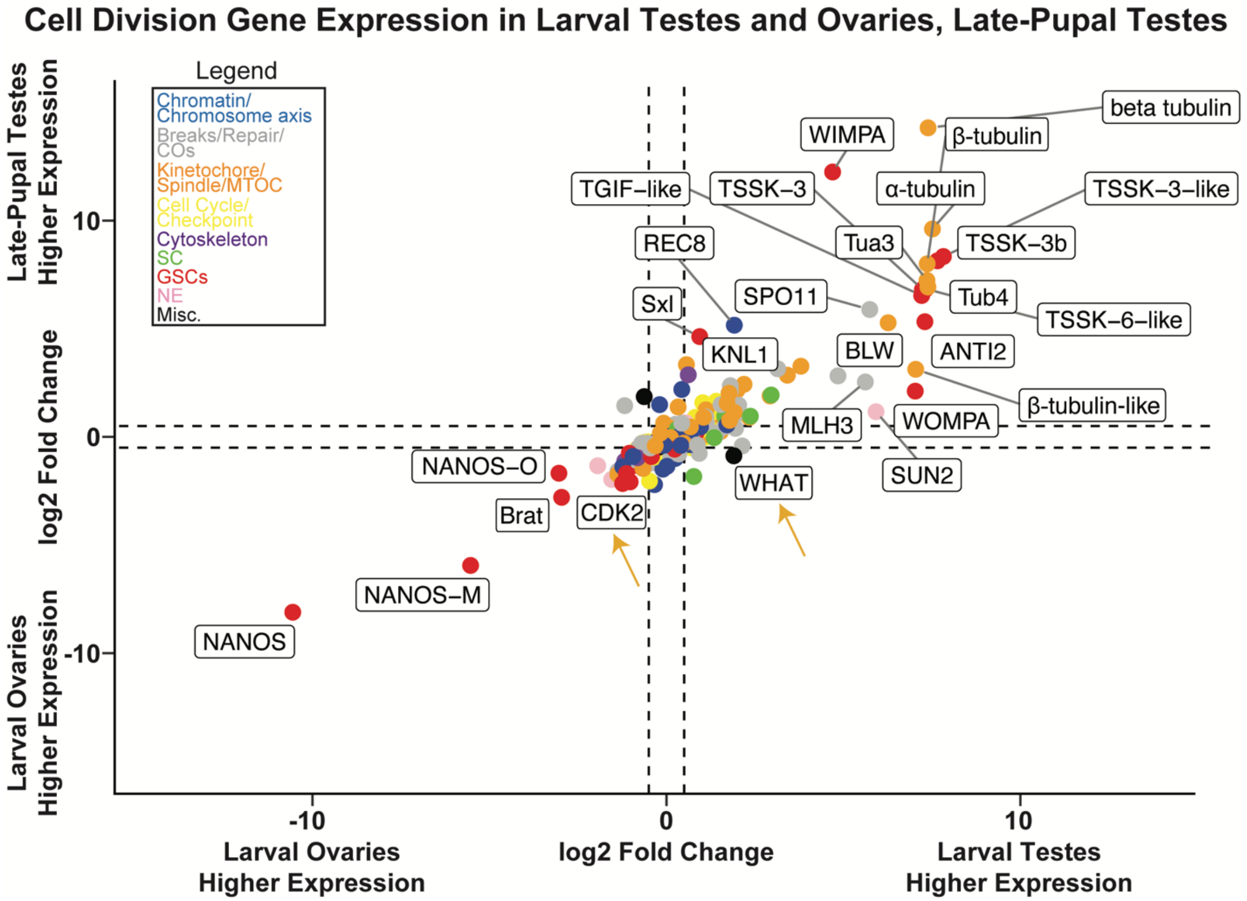

RNA-seq log2 fold change values of cell division genes between WT larval testes and larval ovaries (X-axis) and WT late-pupal testes and WT larval ovaries (Y-axis). Select genes and their cell cycle roles, chromatin/chromosome axis (blue), breaks/repair/COs (gray), kinetochore/spindle/MTOC (orange), cell cycle/checkpoint (yellow), cytoskeleton (purple), synaptonemal complex (SC, green), GSCs (germline stem cells, red), nuclear envelope (NE, pink) and miscellaneous (black) are indicated in the legend. Arrows indicate genes CDK2 and WHAT as discussed in the text, which are more highly expressed in larval ovary than pupal testes

Supplemental Dataset Legends

Dataset S1. Meiosis gene expression in *B. mori* tissues. log2 fold change values and p-adjusted values from differential expression analysis between *Sxl*$^{-}$ late-pupal vs WT late-pupal testes, WT larval vs WT late-pupal testes, and WT larval testes vs WT larval ovaries are listed for each meiotic gene.

Dataset S2. All gene expression in *B. mori* tissues. log2 fold change values and p-adjusted values from differential expression analysis between WT larval testes vs WT larval soma, WT late-pupal testes vs WT late-pupal soma, *Sxl*$^{-}$ late-pupal vs WT late-pupal testes, WT larval testes vs WT larval ovaries, and WT larval vs WT late-pupal testes are listed for each gene.

Dataset S3. Significant GO terms for larval enriched gene expression. Significant GO terms for significantly enriched gene expression in WT larval testes vs WT late-pupal testes.

Dataset S4. BAT1 psiBLAST and HHpred hits.

Dataset S5. WHAT psiBLAST and HHpred hits.

Dataset S6. Gene level hierarchical cluster analysis. Scaled mean RPKM values and cluster identity numbers for each gene in the cluster analysis.

Dataset S7. Significant GO terms for cluster analysis. Significant GO terms for genes belonging to each cluster from the hierarchical cluster analysis.

Dataset S8. Putative transcription factors. log2 fold change values and p-adjusted values from differential expression analysis between *Sxl*$^{-}$ late-pupal vs WT larval testes, WT larval vs WT late-pupal testes, and *Sxl*$^{-}$ late-pupal vs WT late-pupal testes are listed for each putative transcription factor.

Dataset S9. Antibodies and probes used in this study.

Appendix S1. Detailed Materials and Methods.

Rearing of silkworms: Wildtype silkworm embryos were obtained from Educational Science https://www.educationalscience.com, or larvae were purchased from FramsChams Chameleon Breeders (https://framschams.com/collections/silkworms). Silkworm embryos were hatched in a petri dish at room temperature (RT). Larvae were fed a diet of fresh mulberry leaves collected in the wild or mulberry chow and feeding boxes were changed regularly. *Sex-Lethal* mutant pupae were obtained from the National Bio-Resource Project (NBRP) of the MEXT, Japan at Kyushu University in Fukuoka, Japan (https://shigen.nig.ac.jp/silkwormbase/about_kaiko.jsp).

Silkworm and spermatogenesis staging.

Silkworm staging

For microscopy: There are 5 instar larval stages, with each stage having a molting. Maturing through 1-5th instars takes between 19 to 25 days (1). 5th instar larvae were determined based on their time since hatching and large size. The 5th instar larvae are significantly larger in diameter and length than the previous instars, with maximum pigmentation. Mature silkworm stage proceeds 5th instar stage and occurs right before cocooning. It was determined by a yellowish-translucent color on the ventral side of the silkworm, as well as the silkworms stopping to eat in preparation for cocooning. Pupa stages were determined by cutting a small observation hole in the top of the cocoon. Pre-pupation stage contains a small but normal looking silkworm. After that there is a transition to pupation, where the silkworm will change its appearance to a bulb-like shape that is brown and yellow. Every day after that transition would be marked, hence 'day 1 pupa' translates to seeing a pre-pupa the day before, followed by seeing a pupa the next day.

For genomics: Larval tissues were harvested from late 4th/early 5th instar *B. mori* larvae (>5 cm long), mid-pupae (~5-8 days after pupation), or late-pupae (~11-15 days after pupation, after pigmentation had developed in the eyes and abdomen). Eye and abdomen pigmentation was used as the primary marker for late-pupal development to account for potential delays in development in *Sxl* mutants.

Spermatogenesis staging

<u>Staging and identification of prophase I and metaphase I cells in apyrene spermatocytes</u>: For prophase I, staging was determined based on the presence of semi-diffuse SC lateral elements (SYCP2, SYCP3, or HOP1), and nucleus size. Metaphase I cells were identified based on proximity to prophase I cells and the presence of distinct dot-like compacted chromosomes in semi-aligned or cloud-like organizations.

Identification of meiosis II cells in apyrene spermatocytes: Cells in apyrene testes were determined to be in meiosis II based on the presence of micronuclei that resulted from

the previous meiosis I division, in addition to the small size and number of nuclei in the cysts.

Quantification of spermatocytes throughout development: This was done through the distinctive SYCP2 differences between eupyrene and apyrene spermatocytes, as well as the overall size and shape of nuclei between eupyrene and apyrene spermatocytes using DAPI. SYCP2 appears as distinct foci in apyrene spermatocytes, rather than appearing as threads and therefore making it easily distinguishable from eupyrene spermatocytes. Furthermore, when eupyrene and apyrene spermatocytes are seen side by side, the signal from SYCP2 is much dimmer for apyrene cells, helping to identify them. Similarly, apyrene spermatocytes nuclei are considerably smaller than that of eupyrene spermatocytes, making identification easy using DAPI.

Sperm bundle classification: All sperm bundles were identified based on tubulin staining of flagellar tails. Eupyrene sperm bundles can be clearly distinguished from apyrene bundles based on nuclear morphology. Eupyrene bundles have distinct needle-shaped nuclei near the tip of the flagella. Apyrene sperm bundles, on the other hand, have round or oval, highly organized, nuclei near the beginning or mid-point of the flagellar length. Like WT apyrene bundles, Sxl- sperm bundles (identified by tubulin staining of flagella) lack the characteristic needle-shaped and tightly bundled sperm nuclei seen in eupyrene sperm. Instead, Sxl- sperm harbor disorganized, small nuclei that are punctate or oblong in shape and localize throughout the length of the flagella.

B. mori REC8 identification and antibody generation

To identify *B. mori* REC8, the human REC8 (NP_001041670.1) amino acid sequence was used to search the *B. mori* database in NCBI (taxid:7091) using BLAST. This revealed an uncharacterized protein LOC101737592 isoform X1 and X2 [Bombyx mori] (NCBI Reference Sequence: XP_004929454.1) with high sequence homology in the Rad21_REC8 domain to human and Mouse REC8. DNA for the N-terminal 210 aa of XP_004929454.1 was obtained via artificial synthesis by IDT DNA technologies INC in Iowa. The DNA was cloned in pET-21a and the peptide was expressed and purified using the in-frame HIS tag in the vector and Ni-agarose beads (MilliporeSigma). The purified peptide was used to generate antibodies at Cocalico Biologicals in Denver, PA.

Slide preparation, cell counting, nuclear area size, immunofluorescence, and DNA FISH for Figures 1, 2A-C, S1A and S2:

Testes processing and cryosectioning: Testes from male *B. mori* at the relevant developmental stage were carefully dissected. For preparation of fixed frozen 5th instar testes samples, the testes were placed in 4% formaldehyde in PBS and incubated at RT for 20 min. Following fixation, the testes were washed in 0.1% PBST (PBS + 0.1% Tween-20) 3 times, 10 min each. The testes were then embedded in OCT compound (Tissue-Tek, Sakura, Japan, cat #4583). For fresh frozen preparation of pupal testes,

the samples were promptly embedded in OCT to maintain their structural integrity. The OCT-embedded samples were then snap-frozen on dry ice and stored at −80°C until sectioning.

Cryosectioning was performed using a Cryostat Microtome (Cryostar NX70, ThermoFisher). The cryostat was pre-cooled to −11°C, and the tissue blocks were mounted onto the chuck using OCT with the desired orientation. The blocks were allowed to equilibrate at the cryostat temperature for 30 min to ensure uniform sectioning. Sections of 10 μm thickness were then cut. For fixed frozen testes, sections were collected on Sure Bond Charged Microscope Slides (AVANTIK, cat #SL6332-1) and allowed to air dry for 30 min at RT. For freshly frozen testes, sections were mounted onto pre-chilled Sure Bond Charged Microscope Slides and stored at −80°C for post-fixation before using.

For slides requiring post-fixation, tissue sections on slide were fixed with ice-cold 4% paraformaldehyde (PFA) in PBS for 20 min at RT, followed by three 10 min washes in PBS to remove the fixative. The slides were then transferred to 70% ethanol for 2 hr at −20°C and subsequently incubated overnight in 100% ethanol at −20°C. Afterward, the slides were allowed to air-dry and were then stored at −80°C for future use.

Immunofluorescence (IF) on cryosectioned testes: For IF on testes cryosections, an antigen retrieval step was performed prior to immunostaining where the slides were put in 10 mM sodium citrate buffer (pH 6.0) at 90°C for 20 min. The slides were allowed to cool in solution before being immersed into 0.1% PBST for 5 min. Slides were incubated with blocking solution (0.1% PBST + 0.5% BSA) for at least 2 hr before primary antibodies were applied in 0.1% PBST (see Table S9 for concentration) and incubated at 4°C overnight. Slides were washed in 0.1% PBST three times for 5 min before secondary antibody was applied in 0.1% PBST (Table S9) for 2 hr. This was followed by washing in 0.1% PBST three times for 5 min. Slides were stained with 10 μg/mL of DAPI + Vectorshield mounting media for 20 min at RT.

IF/FISH for telomeres on cryosectioned testes: To perform IF with fluorescence *in situ* hybridization (FISH), sectioned and spread testes were first treated with 4% PFA for 20 min and then washed three times for 5 min with 0.1% PBST. Prior to immunostaining, an antigen retrieval step was performed where the slides were put in 10 mM sodium citrate buffer (pH 6.0) at 90°C for 20 min. The slides were allowed to cool in solution before being immersed into 0.1% PBST for 5 min. Slides were incubated with blocking solution (0.1% PBST + 0.5% BSA) for at least 2 hr before primary antibodies were applied in 0.1% PBST at 1:250 dilution at 4°C overnight. Slides were washed in 0.1% PBST before secondary antibody was applied in 0.1% PBST (Table S9) overnight at 4°C (2). This was followed by washing in 0.1% PBST three times for 5 min. RNA was digested with 200 μg/mL of RNAse in PBS for 2 hr at 37°C, and slides were rinsed with PBS two times. Slides were dehydrated with ice-cold 100% methanol for 30 min, and the slides were air dried for 1 hr. Telomere probe DNA (100 ng per slide) was mixed with hybridization buffer (50% formamide, 2X SSC, 1% dextran sulfate, 100 μg/mL salmon sperm DNA), applied on slides, and both genomic DNA and probe DNA were denatured simultaneously on a heating block at 80°C for 5 min. Hybridization was performed at 37°C for 16-20 hr. Slides were washed with 2X SSC for 5 min, 50%

formamide/2X SSC for 15 min at 37°C, 2X SSC for 10 min two times, and 2X SSC/0.1% Triton X for 10 min. Slides were stained with 10 µg/mL of DAPI for 30 min at RT. Slides were mounted with ProLong Gold antifade mountant (Invitrogen, cat #36930).

IF/FISH with Oligopaints on cryosectioned testes: For IF/FISH with Oligopaints, IF was performed as described above, but with additional wash step following the 5 min wash (PBS + 0.1% triton X-100) after antigen retrieval of 15 min wash with 0.5% PBST (PBS + 0.5% triton X-100). This was followed by FISH protocol as previously described (3, 4). Briefly, cryosectioned slides were post-fixed with 4% PFA for 10 min. After this fix, the slides were washed 2X SSCT for 5 min at RT. This was followed by the slides being treated with 2X SSCT/20% formamide for 10 min at RT, 2X SSCT/50% formamide for 10 min at RT, 2X SSCT/50% formamide for 3 min at 92°C, and 2X SSCT/50% formamide for 20 min at 60°C. Primary Oligopaint probes were resuspended in hybridization buffer (10% dextran sulfate/2X SSCT/50% formamide/4% polyvinylsulfonic acid), placed on slides, covered with a coverslip, and sealed with rubber cement, and let to fully dry. After drying, the slides were then denatured for 3 min at 92°C and incubated overnight at 37°C. The next day, coverslips were removed using a razor blade, and slides were washed as follows: 2X SSCT at 60°C for 15 min, 2X SSCT at RT for 15 min, and 0.2X SSC at RT for 5 min. Fluorescently labeled secondary probes were then added to slides, again resuspended in hybridization buffer, covered with a coverslip, and sealed with rubber cement. Slides were incubated at 37°C for 2 hr in a humidified chamber before repeating the above washes. All slides were stained with DAPI, washed twice for 5 min at RT, and mounted in Prolong Glass Antifade Mount.

Imaging and analysis: Images were acquired with a DeltaVision microscopy system (Leica Microsystems) consisting of a 1X70 inverted microscope with a high-resolution CCD camera (2). Images were deconvolved using SoftWoRx v. 7.2.2 (Leica Microsystems Inc.) software. Image analysis was done using Fiji and cropped in Adobe Photoshop. Brightness and contrast were adjusted minimally and uniformly to visualize signals during figure preparation.

Telomere number was analyzed by the following method: Cell segmentation was performed using Cellpose based on DAPI signal, and a filtering step was implemented to exclude artifacts, debris, and poorly segmented cells. Following segmentation, FISH signal dots within each cell were detected and localized using the Find Maxima function in FIJI, which identifies intensity peaks corresponding to individual FISH signals based on user-defined noise tolerance and prominence thresholds. The number of FISH dots was then counted for each cell to measure the difference.

Nuclear area determination: Area of the spermatocyte nuclei was calculated using DAPI with all sections of the z-stack used. In ImageJ/Fiji, the background was subtracted using a rolling ball radius of 200.0 pixels with no extra settings checked. Next a Gaussian blur radius of 2.00 was used. The image was then put through the default threshold function to highlight the area of DAPI, and area size overlay was adjusted to the same size as the DAPI signal. Next a size threshold was established using the Analyze Particle function, and a size of 10 μm^2 to 50 μm^2 was used for most cell sizes for eupyrene and apyrene. The Measure function was then used to calculate the area for each cell in the image.

Volume and Sphericity: Deconvolved images were segmented in 3D and analyzed using a modified version of the TANGO 3D-segmentation plug-in for ImageJ as previously described (4, 5), using either the 'Hysteresis' or 'Spot Detector 3D' algorithms. Statistical analyses were performed in Prism 10 software (GraphPad).

Slide preparation, Immunofluorescence, and DNA FISH for Figure S1C-D:

IF/FISH was performed on cytospin-based spermatocyte spreads. Here, testes were dissected out and placed in 0.5% sodium citrate, where they were dissociated by gentle pressure with a pestle. Loose germline cysts were then incubated in the sodium citrate for 10 min. Cysts were spun onto slides using a Cytospin 4 (Thermo) with the following settings: 500 x g, 5 min, max acceleration. Following spinning, cells were fixed to slides with 4% PFA for 10 min, and washed thrice with 0.1% PBS-T. Cells were permeabilized with 0.5% Triton X-100 in PBS for 15 min, blocked in 5% BSA for 1 hr at RT, and then incubated with primary antibodies overnight. The next day, slides were washed thrice in PBS-T, incubated with secondary antibodies, washed again, then post-fixed before proceeding to DNA FISH. The DNA FISH protocol was exactly as previously described (6).

Slides were imaged on a Leica DMi6000 inverted wide-field fluorescence microscope equipped with an APO 63x/1.40 Oil objective (Leica), Leica DFC9000 sCMOS Monochrome Camera, EL6000 light source, DAPI/FITC/CY3/CY5 filter cubes, and LasX software. Images were deconvolved using Huygens deconvolution software (SVI) and tiffs were generated with imageJ.

Slide preparation and Immunofluorescence Figures 2D, 2F-H, 3, S3-S5, S9B-C:

For IF on manual spermatocyte spreads, testes were harvested from 5th instar *B. mori* larvae (>5 cm long) or mid-stage pupae (~5-8 days after pupation) in 1X PBS, washed twice in hypotonic buffer (0.1 M sucrose, 5 mM EDTA, 0.1 mM phenylmethylsulfonyl fluoride (PMSF)), and incubated in fresh hypotonic buffer for 30 min on ice. Testes were then homogenized in the hypotonic buffer with a pestle and the spermatocyte suspension incubated for 30 min on ice. Spreads were fixed (1.6% PFA, 0.15% Triton-X, 0.05 M sucrose, 5 mM EDTA) in a humidified chamber for 3 hr and then air-dried at RT. Slides were then washed in Photoflo for 2 min before proceeding to IF. For IF of SC components, immediately following washing, slides were blocked in 5% milk in 0.1% PBST for 1 hr at RT. For DSN1 and Tubulin staining, cells were first subjected to permeabilizations with 100% methanol at RT for 20 min, then PBS-Triton 0.5% at RT for 15 min before blocking in 2% BSA at RT for 1 hr. For all slides, primary antibodies were diluted in blocking solution to the concentrations listed in Table S9 and incubated on slides under parafilm coverslips overnight at 4°C. Slides were then washed thrice in 0.1% PBST before adding secondary antibodies for a 1 to 2 hr RT incubation. After washing off secondary antibodies, slides were DAPI stained, washed thrice in 0.1% PBST, mounted in Slow-Fade or Prolong Diamond, and sealed with nail polish before imaging.

For IF on whole mount testes, testes were harvested from 5th instar *B. mori* larvae (>5 cm long), late-stage pupae (~11-15 days after pupation), and adult in ice-cold 1X PBS. Testes were fixed in 4% PFA in PBST for 15 min. Testes were then washed twice in 0.1% PBST before rinsing with 1x PBS. Testes were first subjected to permeabilizations with 100% methanol on ice for 10 min, then 0.5% PBST at RT for 15 min before blocking in 2% BSA at RT for 1 hr. Tubulin primary antibodies were diluted in blocking solution to the concentrations listed in Table S9 and incubated overnight at 4°C on a nutator. Testes were then washed thrice in 0.1% PBST before adding secondary antibodies for 2 hr RT incubation with rotation. After washing off secondary antibodies, testes were DAPI stained, washed thrice in 0.1% PBST, mounted in Prolong Diamond on slides with "spacers". Slides were left to cure at RT for at least 24 hr and then sealed with nail polish before imaging.

Images in Figures S3 and S4 were acquired on a Leica DMi8 widefield inverted fluorescence microscope equipped with APO 63x/1.40 CS2 oil objective (Leica), K8 CMOS Monochrome Camera, LED8 light source, and with DAPI/FITC/CY3/ CY5/CY7 filter cubes. All images were processed using the LasX software with Leica Thunder Deconvolution and tiffs were created in ImageJ. Confocal images in Figures 2, 3, and S5 were acquired on a Leica Stellaris 8 Resonant Scanning Confocal with 4 HyD S detectors and 1 HyD X SP detector, and SuperZ Galvo stage. Confocal images were post-processed with Huygens X11 Deconvolution software (SVI).

RNA-seq: Tissue collection, library generation, and analysis

Gonads and somatic tissues (whole carcass minus gonads) for RNA-seq were harvested from WT *B. mori* males and females, staged as described above. *Sxl*⁻ late-pupal testes and somatic tissues were also collected (~11-15 days after pupation, after pigmentation had developed in the eyes and abdomen). All tissues were homogenized in TRIzol and total RNA was isolated using the Direct-zol RNA Miniprep Plus Kit (Zymo Research, Tustin, CA) following manufacturer's instructions. rRNA depleted RNA libraries were generated using Zymo-Seq RiboFree Total RNA Library Kit (Zymo Research) and indexed with Zymo-Seq Unique Dual Index (UDI) Primer Plate (Zymo Research) according to the manufacturer's instructions. Libraries were sequenced on a NovaSeq 6000 SP200 flow cell (100 bp paired-end sequencing, Illumina, San Diego, CA). Biological replicates were done in quadruplicates except for one larval ovary replicate because it was a failed library and therefore removed from the analysis.

RNA-seq reads were mapped to the NCBI *B. mori* genome (NCBI RefSeq assembly:GCF_030269925.1) (7) with HISAT2 software (8) (-k 1 --rna-strandness RF --dta). Mapped reads were then sorted with samtools (9) and then HTseq-count (10) (-f bam -i gene_id -s reverse -r pos) was used to count the number of reads mapping to each gene utilizing the NCBI *B. mori* GTF annotation file (NCBI RefSeq GTF:GCF_030269925.1). DeSeq2 (11) was used to determine differentially expressed genes between developmental timepoints, tissues, or genotypes. A log2 fold change value greater than 0.5 or less than -0.5 with a p-adjusted value less than 0.05 were the cutoff parameters for determining if genes were differentially expressed or not between

samples. For *de novo* transcript assembly, stringtie software (12) was used to create individual GTF files from each RNA-seq replicate's bam file using the NCBI *B. mori* GTF file as a reference annotation. Individual GTF files were then merged with the stringtie merge function to create a single *de novo* transcript assembly. BigWig tracks were generated with Deeptools software bamCoverage (13) (-bs 5 --effectiveGenomeSize 530000000 --normalizeUsing BPM). Gene level tracks were visualized with UCSC Genome Browser (14). ggplot2 was used to generate MA plots (15) and pheatmap was used to generate heatmaps (16). RNA-seq replicate PCA analysis was performed with R software (17) plotPCA() and euclidean distances for hierarchical clustering analysis was performed with the dist() function. Gene level hierarchical clustering analysis for all tissues was performed with the kmeans() function (k=5). Gene ontology analysis on differentially expressed genes and clusters was performed with g:Profiler's g:GOSt functional profiling software (18). The *B. mori* (*B. mori* (Domestic silkworm, p50T)) organism was used as the background gene set and a p value threshold of 0.05 was used to determine functional gene ontology terms.

ATAC-seq: Tissue collection, library generation, and analysis

Tissues for ATAC-seq were dissected from *B. mori* wildtype late instar larval testes, mid-pupal testes, late-pupal testes, and *B. mori* Sxl late-pupal testes in ice-cold PBS. Testes were thoroughly homogenized with a pestle in a 1.5 mL tube. ATAC-seq digestion and library generation was performed using the ATAC-seq Kit (Active Motif, catalog 53150), following manufacturer's protocol with slight modification, i.e. tagmentation reaction with less volume (5 µL) of Assembled Transposomes. QC of ATAC libraries was performed using Qubit and TapeStation. Libraries were sequenced on a Illumina NovaSeq 6000 with SP100 flow cell (50 bp paired-end sequencing). Samples were done in quadruplicates.

ATAC-seq reads were mapped to the NCBI *B. mori* genome (NCBI RefSeq assembly:GCF_030269925.1) (82) with HISAT2 software (83) (-k 1 --no-spliced-alignment -X 900). Mapped reads were then sorted with samtools (84) and duplicate reads were removed with samtools markdup. Significant ATAC-seq peaks for each tissue were called with Macs3 software (-f BAMPE -g 1.2e8 -q 0.0001)(PMID: 18798982). BigWig tracks were generated with Deeptools software bamCoverage (88) (-bs 5 --effectiveGenomeSize 530000000 --normalizeUsing BPM).

Protein homology searches and cell division gene validation

For annotation of genes potentially involved in cell division (Table S2), we collated a list containing: 1. cell cycle regulators (APC/C, cyclins, CDKs), 2. general germ cell and germline stem cell factors with a focus on those found in *D. melanogaster* and *B. mori* (e.g. piRNA pathway genes, spermatogenesis, stem cell markers), 3. kinetochore proteins (including spindle assembly checkpoint (SAC)), 4. chromatin architecture proteins (e.g. insulators, cohesin, condensin and SMC5/6), 5. known conserved meiotic proteins (19), and 6. an updated version of the genes from the "meiotic toolkit" (2). Genes in groups 5 and 6 defined above include genes involved in DNA double strand break formation and repair (DSB), homologous recombination (HR), synaptonemal

complex formation (SC), pro-crossover factors (CO), and a number of meiotic kinases.

To search for orthologs, we employed a multi-layered strategy. We first selected relevant Hidden Markov Models (HMMs) of orthologs of our gene set from the EggNOG (KOGs/ENOGs – EggNOG mapper v2) (20) and Panther (21) database using the automated annotations in Uniprot (22) for both *H. sapiens* and *D. melanogaster*. In addition, we collected HMMs from previous efforts to detect specific gene families for kinetochores (23), chromosomal architecture

Candidates were subsequently recovered using HMMsearch from the HMMER package with standard settings (25). Significant hits were cross-validated for orthology by performing reciprocal similarity searches between *B. mori* and *H. sapiens*/*D. melanogaster* via direct BLASTp using the bidirectional-best-hit principle (26). For large gene families with many paralogs (e.g. kinases), we generated phylogenetic trees to delineate orthologs using the IQ-tree webserver (standard settings including model selection and 1000 UF bootstraps) (27).

In case no homology links could be established through our standard approach, we used three remote homology detection routes: (1) HMMsearch without heuristic filtering ('–max' setting) that occasionally finds remote orthologs at the cost of high computational burden; (2) iterative HMM searches using jackhmmer via the online webserver (25) in both directions (starting from the *Bombyx* or human/fly sequences) focusing on animal species, considering candidates that would be clearly part of single gene family; and (3) reciprocal HMM-vs-HMM profile searches with HHpred using *D. melanogaster* and *H. sapiens* and PFAM pre-calculated HMMs (28). If none of these methods provided any significantly similar candidates, they were not included in our analyses. For notes on gene discovery see Table S2.

Additional PsiBLAST (29), HMMER, and HHpred searches for BAT1 and WHAT were performed using the MPI Bioinformatics Toolkit (30, 31) (https://toolkit.tuebingen.mpg.de/) and the EMBL-EBI HMMER web server (32) (https://www.ebi.ac.uk/Tools/hmmer/home). Phylip Neighbor-joining tree of putative WHAT-like proteins was created with clustalw2 (33) without distance correction based on a multiple sequence alignment (MSA) generated with MUSCLE (34). MSA schematic was generated with Geneious Prime (Dotmatics).

References

1. Tazima, Y., *The silkworm: an important laboratory tool.* (Kodansha Ltd., 1978).

2. Y. Xiang, *et al.*, A molecular cell biology toolkit for the study of meiosis in the silkworm Bombyx mori. *G3 (Bethesda)* **13** (2023).

3. L. F. Rosin, J. J. Gil, I. A. Drinnenberg, E. P. Lei, Oligopaint DNA FISH reveals telomere-based meiotic pairing dynamics in the silkworm, Bombyx mori. *PLoS Genet* **17**, e1009700 (2021).

4. L. F. Rosin, S. C. Nguyen, E. F. Joyce, Condensin II drives large-scale

folding and spatial partitioning of interphase chromosomes in Drosophila nuclei. *PLOS Genetics* **14**, e1007393 (2018).

5. J. Ollion, J. Cochennec, F. Loll, C. Escudé, T. Boudier, TANGO: a generic tool for high-throughput 3D image analysis for studying nuclear organization. *Bioinformatics* **29**, 1840–1841 (2013).

6. L. F. Rosin, D. Chen, Y. Chen, E. P. Lei, Dosage compensation in Bombyx mori is achieved by partial repression of both Z chromosomes in males. *Proc Natl Acad Sci U S A* **119**, e2113374119 (2022).

7. J. Lee, T. Fujimoto, K. Sahara, A. Toyoda, T. Shimada, Comprehensive genome annotation of Bombyx mori p50ma strain, a newly developed standard strain. *Sci Data* **12**, 359 (2025).

8. D. Kim, J. M. Paggi, C. Park, C. Bennett, S. L. Salzberg, Graph-based genome alignment and genotyping with HISAT2 and HISAT-genotype. *Nat Biotechnol* **37**, 907–915 (2019).

9. P. Danecek, *et al.*, Twelve years of SAMtools and BCFtools. *Gigascience* **10** (2021).

10. S. Anders, P. T. Pyl, W. Huber, HTSeq--a Python framework to work with high-throughput sequencing data. *Bioinformatics* **31**, 166–169 (2015).

11. M. I. Love, W. Huber, S. Anders, Moderated estimation of fold change and dispersion for RNA-seq data with DESeq2. *Genome Biol* **15**, 550 (2014).

12. A. Shumate, B. Wong, G. Pertea, M. Pertea, Improved transcriptome assembly using a hybrid of long and short reads with StringTie. *PLoS Comput Biol* **18**, e1009730 (2022).

13. F. Ramírez, *et al.*, deepTools2: a next generation web server for deep-sequencing data analysis. *Nucleic Acids Res* **44**, W160-165 (2016).

14. W. J. Kent, *et al.*, The human genome browser at UCSC. *Genome Res* **12**, 996–1006 (2002).

15. H. Wickham, *ggplot2: Elegant Graphics for Data Analysis* (Springer-Verlag New York, 2016).

16. R. Kolde, pheatmap: Pretty Heatmaps. (2025). Deposited 2025.

17. R Core Team, R: A language and environment for statistical computing. (2021). Deposited 2021.

18. L. Kolberg, *et al.*, g:Profiler—interoperable web service for functional enrichment analysis and gene identifier mapping (2023 update). *Nucleic Acids Research* **51**, W207–W212 (2023).

19. S.-B. Malik, A. W. Pightling, L. M. Stefaniak, A. M. Schurko, J. M. J. Logsdon, An expanded inventory of conserved meiotic genes provides evidence for sex in Trichomonas vaginalis. *PLoS One* **3**, e2879 (2007).

20. J. Huerta-Cepas, *et al.*, eggNOG 5.0: a hierarchical, functionally and phylogenetically annotated orthology resource based on 5090 organisms and 2502 viruses. *Nucleic Acids Research* **47**, D309–D314 (2019).

21. P. D. Thomas, *et al.*, PANTHER: Making genome-scale phylogenetics accessible to all. *Protein Sci* **31**, 8–22 (2022).

22. UniProt Consortium, UniProt: the universal protein knowledgebase in 2021. *Nucleic Acids Res* **49**, D480–D489 (2021).

23. J. J. van Hooff, E. Tromer, L. M. van Wijk, B. Snel, G. J. Kops, Evolutionary dynamics of the kinetochore network in eukaryotes as revealed by comparative genomics. *EMBO Rep* **18**, 1559–1571 (2017).

24. J. J. E. van Hooff, M. W. D. Raas, E. C. Tromer, L. Eme, Repeated duplications and losses shaped SMC complex evolution from archaeal ancestors to modern eukaryotes. *Cell Rep* **44**, 115855 (2025).

25. S. C. Potter, *et al.*, HMMER web server: 2018 update. *Nucleic Acids Res* **46**, W200–W204 (2018).

26. S. F. Altschul, W. Gish, W. Miller, E. W. Myers, D. J. Lipman, Basic local alignment search tool. *J Mol Biol* **215**, 403–410 (1990).

27. J. Trifinopoulos, L.-T. Nguyen, A. von Haeseler, B. Q. Minh, W-IQ-TREE: a fast online phylogenetic tool for maximum likelihood analysis. *Nucleic Acids Res* **44**, W232-235 (2016).

28. J. Söding, A. Biegert, A. N. Lupas, The HHpred interactive server for protein homology detection and structure prediction. *Nucleic Acids Res* **33**, W244-248 (2005).

29. S. F. Altschul, *et al.*, Gapped BLAST and PSI-BLAST: a new generation of protein database search programs. *Nucleic Acids Research* **25**, 3389–3402 (1997).

30. L. Zimmermann, *et al.*, A Completely Reimplemented MPI Bioinformatics Toolkit with a New HHpred Server at its Core. *Journal of Molecular Biology* **430**, 2237–2243 (2018).

31. F. Gabler, *et al.*, Protein Sequence Analysis Using the MPI Bioinformatics Toolkit. *Curr Protoc Bioinformatics* **72**, e108 (2020).

32. F. Madeira, *et al.*, The EMBL-EBI Job Dispatcher sequence analysis tools framework in 2024. *Nucleic Acids Res* **52**, W521–W525 (2024).

33. M. A. Larkin, *et al.*, Clustal W and Clustal X version 2.0. *Bioinformatics* **23**, 2947–2948 (2007).

34. R. C. Edgar, MUSCLE: multiple sequence alignment with high accuracy and high throughput. *Nucleic Acids Res* **32**, 1792–1797 (2004). , and the meiotic toolkits mentioned above.
